# Computational Studies of Rubber Ozonation Explain the Effectiveness of 6PPD as an Antidegradant and the Mechanism of Its Quinone Formation

**DOI:** 10.1021/acs.est.2c08717

**Published:** 2023-03-24

**Authors:** Elliot Rossomme, William M. Hart-Cooper, William J. Orts, Colleen M. McMahan, Martin Head-Gordon

**Affiliations:** † Bioproducts Research Unit, Agricultural Research Service, U.S. Department of Agriculture, Albany, California 94710, United States; ‡ Berkeley Center for Green Chemistry, University of California, Berkeley, California 94720, United States; ¶ Chemical Sciences Division, Lawrence Berkeley National Laboratory, Berkeley, California 94720, United States; § Department of Chemistry, University of California, Berkeley, California 94720, United States; ∥ Kenneth S. Pitzer Center for Theoretical Chemistry, University of California, Berkeley, California 94720, United States

**Keywords:** 6PPD, ozone, mechanism, quinone, DFT, EDA

## Abstract

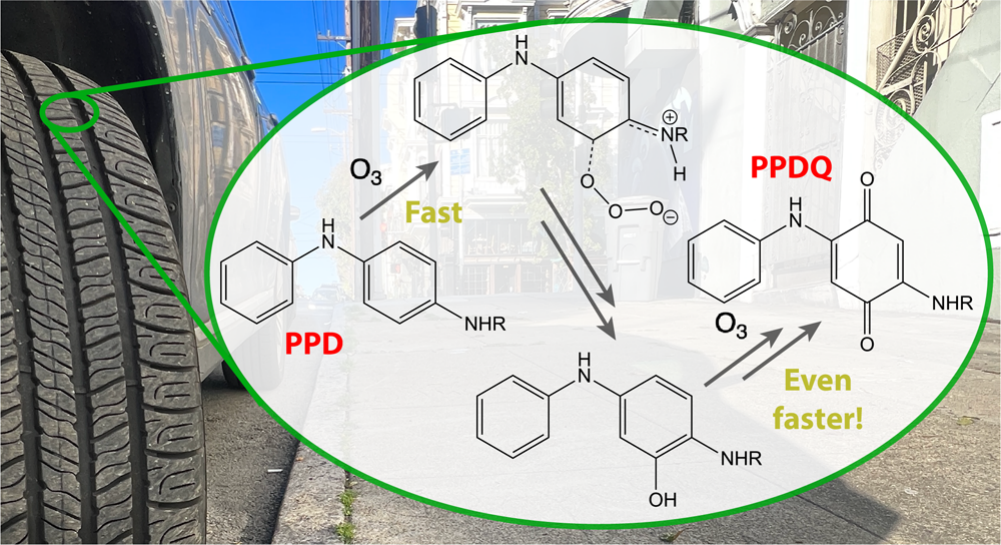

The discovery that the commercial rubber antidegradant 6PPD reacts with ozone (O_3_) to produce a highly toxic quinone (6PPDQ) spurred a significant research effort into nontoxic alternatives. This work has been hampered by lack of a detailed understanding of the mechanism of protection that 6PPD affords rubber compounds against ozone. Herein, we report high-level density functional theory studies into early steps of rubber and PPD (*p*-phenylenediamine) ozonation, identifying key steps that contribute to the antiozonant activity of PPDs. In this, we establish that our density functional theory approach can achieve chemical accuracy for many ozonation reactions, which are notoriously difficult to model. Using adiabatic energy decomposition analysis, we examine and dispel the notion that one-electron charge transfer initiates ozonation in these systems, as is sometimes argued. Instead, we find direct interaction between O_3_ and the PPD aromatic ring is kinetically accessible and that this motif is more significant than interactions with PPD nitrogens. The former pathway results in a hydroxylated PPD intermediate, which reacts further with O_3_ to afford 6PPD hydroquinone and, ultimately, 6PPDQ. This mechanism directly links the toxicity of 6PPDQ to the antiozonant function of 6PPD. These results have significant implications for development of alternative antiozonants, which are discussed.

## Abstract 2

Mechanistic DFT computations explain the reactivity of 6PPD toward ozone and directly link PPD function to toxicity through quinone formation.

## 1 Introduction

Rubber tires are essential across various sectors, including transportation and agriculture. Indeed, tire manufacturers produced 19 million tons of rubber in 2019, and continued global industrialization is expected to increase tire demand, requiring nearly 23 million tons annually by 2024.^1^ Maximization of the longevity of tires is a form of sustainability, reducing the annual flow of tires to landfills and other waste streams. This and other pressures have led the development of highly effective rubber additives that protect rubber from degradation during manufacture and use,^2^ most notably *p*-phenylenediamines (PPDs).^3,4^ Among these, 6PPD (*N*-(1,3-dimethylbutyl)-*N*′-phenyl-*p*-phenylenediamine) in particular has gained ubiquity in the tire industry and is included at 0.5–1.5 wt % in standard formulations.^2^ As a result, annual U.S. consumption of 6PPD ranges from 50 to 100 million tons, the majority of which is used in tires.^5^

Despite widespread use, PPD additives are known to aggravate various toxicity end points for both human and environmental health.^6−11^ 6PPD in particular has recently gained notoriety due to the extreme aquatic toxicity of its quinone transformation product (6PPDQ) to coho salmon^12−14^ and other aquatic species.^15−17^ Though unknown for decades, 6PPDQ is produced in nearly 10% molar yield upon ozonation of 6PPD.^18^ While work is ongoing, this process is believed to occur at the surface of tires and tread wear particles^18^ before 6PPD, 6PPDQ, and other transformation products enter water systems through roadway runoff in storms.^19,20^

Replacement of 6PPD poses a formidable challenge as rubber compounds are susceptible to attack from numerous reactive species—peroxyl radicals, alkyl radicals, ozone—and 6PPD protects rubber compounds from each of these degradation pathways.^21−23^ Degradation due to peroxyl and alkyl radicals is relatively well-characterized,^2^ and research into alternative antioxidants was underway well before the discovery of 6PPDQ and its toxicity.^24−34^ The ozonation chemistry of PPDs and the development of safer antiozonants have been more elusive. Broadly speaking, it is believed that 6PPD protects tires in two distinct but overlapping ways:^35^ (1) kinetic scavenging that consumes O_3_ at the tire surface before it is able to react with the rubber^3,36,37^ and (2) subsequent formation of a protective film that provides a mechanical barrier against O_3_.^36,38,39^ Not observed in all PPDs,^37^ these protective films are comprised of PPD reaction products,^38−40^ although details are poorly understood. Reinvestigation of the mechanism of 6PPD ozonation is therefore imperative to understanding the kinetics of its ability to scavenge ozone, the likely products that lead to film formation, and the pathway to 6PPD quinone.

Herein, we report the first investigation into the mechanism of PPD ozonation since the discovery of 6PPDQ.^12^ Others have laid seeds for this work through detection of potential intermediates^41,42^ and developing broad-strokes proposals of the pathway.^12,41,42^ We continue this work through high-level computational analysis of the ozonation pathways of PPDs. Comparison of the barrier heights for various PPD ozonation pathways demonstrates that the route to the quinone is uniquely accessible, linking the toxicity of PPDs directly to their function as antiozonants. Throughout this study, mechanistic proposals are derived from ozonation mechanisms in related systems (Section 1.1), and our computational protocol follows best practices for O_3_ modeling (Section 1.2). We benchmark our methodology against existing methods (Section 3.1) before presenting mechanistic results for ozonation of a rubber surrogate (Section 3.3) and PPDs (Section 3.4). We conclude (Section 3.5) by discussing the implications of this work for development of alternatives to 6PPD.

### 1.1 Ozonation Mechanisms

While the ozonation chemistry of PPDs is underexplored, the literature contains a vast body of work on ozonation reactions in similar systems. Recent reviews and monographs provide a thorough treatment of this chemistry in a wide array of systems.^43,44^ Here, we provide an overview of critical aspects of ozonation chemistry in alkenes, amines, and aromatic systems, which are all relevant to understanding the reactions of O_3_ with rubber systems and/or PPDs.

#### Alkenes

Generally speaking, ozone reacts with unsaturated systems to produce scission products of the parent substrate.^44^ The overall reaction pathway is uncontroversial: ozone adds to olefins to form so-called primary ozonides, which may decompose or rearrange to form (secondary) ozonides, ultimately resulting in scission of the original olefin into two carbonyl products. This pathway explains the degradation of rubber upon exposure to ozone.^37,38,45^

Despite this consensus, two distinct proposals for the mechanism of primary ozonide formation have gained popularity: a concerted addition across the double bond (Criegee mechanism)^46^ and a stepwise addition, where ozone reacts with an olefin to form a biradical intermediate which may subsequently collapse to the ozonide (DeMore mechanism).^47^ Research increasingly indicates that the Criegee mechanism prevails in simple (i.e., less substituted, electronically symmetric) systems,^48−51^ while both steric and electronic effects of olefin substituents increase the activity of DeMore channels.^49,52,53^ Recent theoretical work indicates that solvent effects can make stepwise mechanisms dominant in systems where they otherwise would not be.^54^

#### Amines

The reactivity between amines and O_3_ is generally understood as a nucleophilic attack of N toward O_3_,^55−57^ and this pathway is particularly important in tertiary amines.^58,59^ Depending on the substitution pattern of the parent amine, reaction products may include *N*-oxides, nitrones, hydroxylamines, nitroso-alkanes, nitroalkanes, and dealkylated amines.^57,59−61^ Amines may also react with ozone through insertion into the N–H bond.^62^ The N lone pair plays a critical role in amine reactivity toward O_3_, such that more highly substituted aliphatic amines exhibit increased O_3_ reactivity.^57^ By the same token, these reactions are highly sensitive to pH, as amine protonation eliminates the reaction channel.^59,63^ Even in the most favorable systems and conditions, high barrier heights for N–O bond formations^62^ can limit these reactions relative to other available ozonation pathways.^56^

#### Aromatics

Reference to the ozonation_3_ chemistry of aromatic systems like anilines and phenols also enhances our understanding of its reactivity in PPDs. In both types of systems, ozonation reactions produce manifold products, and a broad array of mechanistic pathways has been suggested.^64−68^ Motifs common to the preceding functional groups are present here as well: primary ozonide formation,^68,69^ nucleophilic attack of ozone from heteroatoms like N^67^ or the aromatic ring,^66,70^ and ring cleavage reactions^65,68,69^ have all been reported in aromatic systems. These initial ozonation steps often produce intermediates that are themselves reactive toward O_3_, complicating reaction pathways.^60^ Electron donating groups activate reactions between aromatic carbons and O_3_, resulting in highly oxidized aromatic products like quinones.^66,68,69,71^

#### PPDs

Historically, work on PPD ozonation has emphasized formation of N–O oxides, analogous to ozonation of amines, resulting in dinitrone products for PPD ozonation.^37,40,72−74^ In the case of 6PPD, this assignment was recently repudiated on the basis of two-dimensional nuclear magnetic resonance spectroscopy,^12^ and quinones are now understood to form instead,^42,75^ representing nearly 10% of the product distribution for 6PPD.^18^ Beyond this, a great number of reaction products have been determined experimentally,^37,40−42,72^ though mechanistic details of these transformations are unclear. A few recent studies have emphasized the formation of quinone diimines (QDIs) in the various ozonation pathways of PPDs, including the formation of 6PPDQ.^41,42,73^ Even still, the stepwise mechanism of C–O bond formation in PPDs is unexplored to the best of our knowledge, despite the ultimate necessity of this motif in the production of 6PPDQ.

#### Charge Transfer Mechanisms

**Figure 1:**
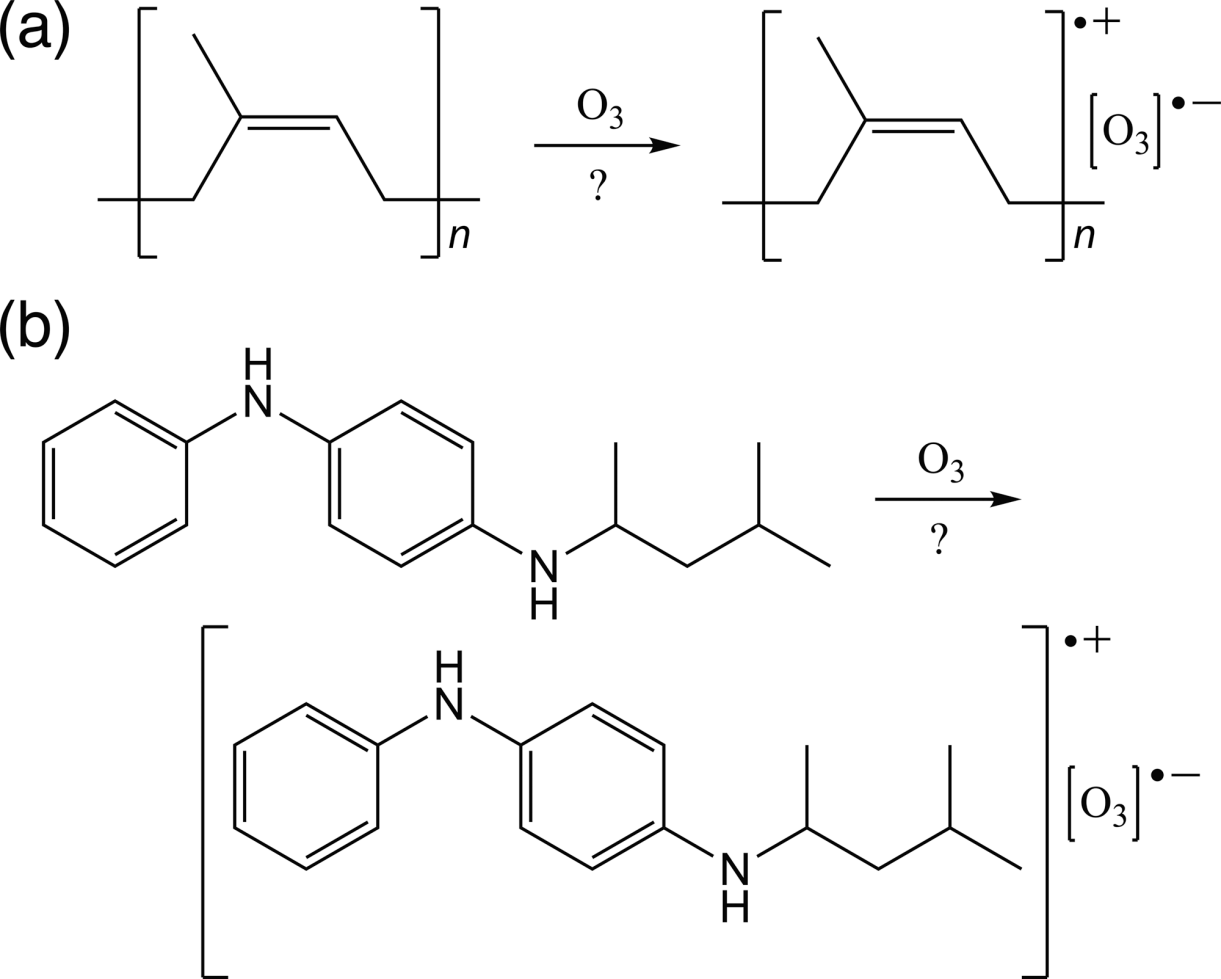
Ozone charge transfer complexes for (a) natural rubber and (b) 6PPD have been proposed by a number of authors (see text).

For each of the classes of reactions discussed above, a number of authors argue that ozonation reactions are initiated by one-electron charge transfer (CT) to form biradical, zwitterionic prereaction complexes (Figure 1). Examples for alkenes,^76−79^ amines,^55−57,59,80^ aromatics,^67,69^ and PPDs^73,74,81,82^ can be found at the indicated references. Such proposals are defended on the basis of a strong correlation between substrate ionization potential and O_3_ reaction rates,^74,76^ electron spin resonance (ESR) spectra that indicate the presence of radicals in the reaction mixture,^73^ or details of product distributions.^59,79^ Some have expressed skepticism about these CT intermediates, which have not been isolated experimentally, though theoretical literature analyzing such proposals is sparse.^4,57,83^ Within the CT mechanism, one-electron transfer is taken to be the rate-determining step of ozonation, so the existence of these complexes for olefins and PPDs is of present interest.

### 1.2 Modeling Ozone Chemistry

Accurate modeling of the electronic structure of ozone (O_3_) is a formidable challenge in quantum chemistry, and many generally reliable methods have been shown to yield (sometimes catastrophically) bad predictions for ozonation chemistry.^84^ Beginning with some of the earliest theoretical work, O_3_ was generally accepted to have a biradical ground state,^85,86^ though subsequent results called this into question. At least one multireference study indicated that the ground state of O_3_ is a “regular” (i.e., closed-shell) singlet, with authors arguing that the electronic structure of O_3_ can be completely captured without reference to biradical character.^87^ Ozone is a difficult modeling problem because the truth lies somewhere between these two extremes, and a number of researchers have quantified the degree of biradical character in O_3_, with values ranging from 16–49%.^88−92^ Regardless of the exact value, it is clear that O_3_ is a genuinely multireference (MR) system. As a result, much of the highest quality theoretical literature on O_3_ chemistry resorts to one of a number of MR schemes.^49,93^

Nevertheless, many single-reference (SR) approaches to O_3_ modeling have been reported.^50,62,94−103^ Errors in these predictions can be substantial. As Wheeler et al. have noted, generally respectable SR methodologies predict barrier heights with discrepancies in excess of 10 kcal mol^–1^ for small systems like C_2_H_2_ and C_2_H_4_, and even the CCSD(T) (coupled-cluster with single, double, and perturbative triple excitations) method gives unsatisfactory results.^99^ To take just one relevant example, these high-level methods predict divergent results for the initial step of O_3_ addition to olefins.^49−51^ In order to systematically approach the fully correlated limit, inclusion of explicit triple and perturbative quadruple excitations (the CCSDT(Q) method) is necessary.^62,99,104^ While Trogolo et al. report results of this quality for a number of small systems,^62^ the  scaling of this method excludes its use for the present systems.

Instead, systems of present interest require treatment with either less computationally demanding wave function approaches or density functional approximations (DFAs). A number of DFA treatments of ozone chemistry have been reported.^57,62,70,97,98^ Generally speaking, however, out-of-the box DFA treatments are unable to predict ozone reaction energies with consistent fidelity,^62^ while more success can be expected for barrier heights.^104^ Hybrid density functionals generally exhibit the best performance for both overall thermodynamics and transition energies.^62^ Spin projection schemes^105^ are known to improve DFA results for related systems,^106^ and this approach has been used to model O_3_ previously.^49,57^ On the other hand, novel low-scaling wave function approaches like regularized orbital-optimized second-order Møller–Plesset perturbation theory (κ-OOMP2) effectively treat radical species in other contexts,^107−109^ though their use for ozonation chemistry has not been previously explored.

## 2 Computational Methods

### Methods of Electron Correlation

In the present study, we consider the ωB97X-V^110^ and ωB97M-V^111^ density functional approximations (DFAs), as well as regularized orbital-optimized second-order Møller–Plesset perturbation theory (κ-OOMP2)^107^ in our modeling of O_3_ reactivity. These density functionals were chosen on the basis that hybrids outperform other classes of DFAs for O_3_ modeling^62^ and that ωB97X-V and ωB97M-V, specifically, have been shown to yield highly accurate predictions for thermochemistry and reaction barrier heights in other contexts.^112^ We also explore the use of κ-OOMP2^107^ as a low-scaling wave function method for modeling ozonation reactions. Though unexplored for O_3_, κ-OOMP2 is able to successfully treat strong correlation in other systems when combined with spin projection,^106^ as discussed further below.

### Exploring Potential Energy Surfaces

Single-point energies were evaluated using either the def2-TZVPP^113^ or def2-QZVPPD^114^ basis set, as indicated in the text below. Exchange–correlation integrals were evaluated using fine-mesh (99,590) Lebedev integration grids.^115,116^ Self-consistent field (SCF) iterations were converged to at least 1 × 10^–8^ a.u. in all cases, and a tighter threshold of 1 × 10^–10^ a.u. was achieved where possible. Unrestricted reference states and stability analysis were used to confirm ground electronic state configurations were obtained. Where included, molecular orbitals were obtained as intrinsic bonding orbitals (IBOs),^117^ using a recently reported implementation.^118^

All geometries utilized in this work were optimized using the ωB97X-V density functional with the def2-TVZPP basis, and stationary points were obtained using gradient and energy thresholds of 3 × 10^–4^ and 1 × 10^–6^ a.u., respectively. Yamaguchi’s approximate spin projection (AP) method^105^ was used to address spin contamination in both DFA and κ-OOMP2 computations (Section S1), and free energies at *T* = 298.15 K were obtained using the quasi rigid rotor-harmonic oscillator scheme with a frequency cutoff of 100 cm^–1^.^119^ While algorithms for structural optimization on spin projection surfaces have been reported,^49,120^ all geometric properties and harmonic vibrational frequencies have been obtained on the (spin-contaminated) singlet energy surfaces. Initial guesses for transition state optimizations were obtained using either the freezing string method^121,122^ or constrained optimizations near the expected transition structure. Harmonic frequencies for each optimized structure were determined through diagonalization of the full Hessian matrix, and stationary points were characterized as local minima or transition states based on the presence or absence of a single imaginary frequency in this analysis. Intrinsic reaction coordinate (IRC) analysis^123,124^ was used to determine which local minima were associated with each transition structure. Coordinates for all such reactant, product, and transition structures are found in the SI. All computations were completed using the Q-Chem package.^125^

### Energy Decomposition Analysis

The adiabatic energy decomposition analysis (EDA) of Head-Gordon and co-workers^126^ was used to evaluate the formation of charge transfer (CT) complexes. Interactions between fragments defined as substrate radical cations (X^.+^) and ozone radical anion (O_3_^.–^) were treated using a hierarchy of mathematical constraints that model frozen electrostatics (FRZ), orbital polarization (POL), and CT contributions to overall binding energies. A full description of this formalism and the components of the different potential energy surfaces may be found elsewhere.^126−128^ Incremental energy contributions are defined as
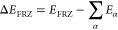
1
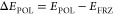
2
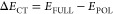
3where *E*_FRZ_, *E*_POL_, and *E*_FULL_ are the minimum energies on the frozen, polarization, and unconstrained PESs, respectively, and *E*_α_ are the electronic energies of the radical ion fragments in unconstrained computations. Free energies were not computed for use with EDA predictions.

For present purposes, it is critical to note that *E*_POL_ contains all terms of the standard (e.g., Born–Oppenheimer, nonrelativistic, *etc.*) physical model but explicitly forbids CT between fragments. By evaluating the energetics of radical ionic fragments on this surface relative to an unconstrained computation, we can determine the feasibility of CT complex formation.

### Choice of Model System

It has long been known that the reactions of O_3_ with both tire rubber and PPDs in tires are surface phenomena.^36^ Indeed, absent the presence of strain, which exposes deeper layers in the rubber to the atmosphere, cracking of the rubber surface does not occur.^129^ In this, experimental evidence demonstrates that the diffusion of PPDs to the rubber surface is rapid enough to prevent rubber ozonation, even given their relatively small concentration.^38,39,130^ We are therefore ultimately interested in understanding these chemistries at the rubber/air interface, rather than deep in the matrix of the tire. Even in the latter case, it is likely the general electrostatic “solvent” effects would be negligible due to the low permittivity of rubber (ε ≈ 3).^131^ We therefore report *in vacuo* energies for all transformations in this work, as these should nicely approximate the chemistry at the interface of environmental interest.

**Figure 2:**
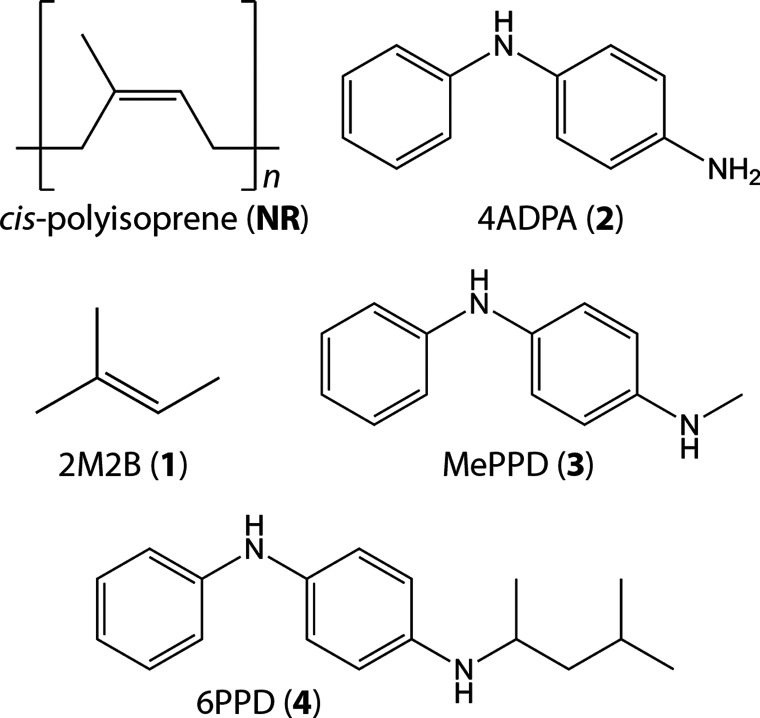
Substrates used in modeling ozone reactivity. Primary exploration of potential energy surfaces was completed using **1** and **2**, as surrogates for **NR** and **4**. Select modeling was completed for compound **3** to better understand experimental results for ozonation kinetics.

The structural optimization procedures outlined above are computationally expensive, bordering on intractability for 6PPD and for large polymeric units of natural rubber (*cis*-polyisoprene, **NR**). We have therefore used surrogate molecules for each of these compounds in our exploration of the potential energy surfaces (PESs) of ozone reaction and the characterization of stationary points. Specifically, we have used 2-methyl-2-butene (2M2B, **1**) and 4-aminodiphenylamine (4ADPA, **2**) as stand-ins for natural rubber and 6PPD, respectively (Figure 2). We have also modeled select key PPD reaction steps using *N*-methyl-*N*′-phenyl-*p*-phenylenediamine (MePPD, **3**) to account for the known effects of *N*-alkylation on PPD ozonation rates.^3,4^ Our *in vacuo* model of reactions of these substrates omits any effects from the tire matrix, but we expect these effects to be small on the basis of the arguments in the preceding paragraph.

## 3 Results and Discussion

### 3.1 Accurate Modeling of Ozone Chemistry

As noted in Section 1.2, accurate modeling of ozonation chemistry is challenging, and model validation is therefore critical. The majority of experimental work on ozonation reactions has been conducted in aqueous or organic solvents, complicating comparison of this work to other systems (e.g., rubber). In the absence of experimental results in an appropriate environment, high-level theoretical results represent the best benchmark for model development. Recent work by Trogolo et al. has provided a nearly exact set of such benchmarks for ozone chemistry,^62^ which we use to assess the model of the present work. Their results consist of CCSDT(Q) energies extrapolated to the complete basis set (CBS) limit for three cycloadditions (C_2_H_4_, C_2_H_2_, HCN), two insertion reactions (HCl, NH_3_), two linear additions (N(CH_3_)_3_ and Br^–^), and three ozone scission reactions (O_3_, N(CH_3_)_3_O_3_, BrO_3_^–^). Benchmark comparisons to these high-level CC reaction energies and barrier heights for the ωB97X-V and ωB97M-V density functionals as well as the κ-OOMP2 approach are found in Table 1, and comparisons for vdW complexes are found in Table S1.

**Table 1:**
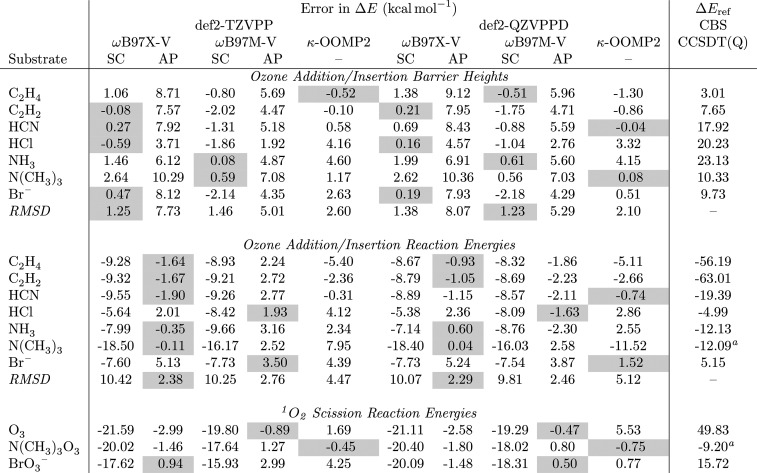
Errors in Spin-Contaminated (SC) and Approximate Projection (AP) Single-Point Energies for Benchmark Ozonation Reactions^b^

a Computed indirectly *via* addition of partial reaction channels. See ref (62) for details.

b For the κ-OOMP2 (κ = 1.45) results, AP is applied to reactions that produce ^1^O_2_, as this is the only species that exhibited spin polarization for this method. Reference values are CCSDT(Q) results extrapolated to the CBS limit obtained from ref (62). Highlighted boxes represent the best performing methodology for a given parameter at a given basis set truncation.

#### Hybrid Density Functionals

As in previous work,^49^ significant spin contamination was found on the singlet DFA potential energy surfaces (PESs) for reactive oxygen species, which we addressed through Yamaguchi’s approximate projection scheme (Section S1).^105^ Comparison of the results from spin contaminated (SC) and approximate projection (AP) energies highlights the complexities of strong correlation in ozone systems. Specifically, these data are conclusive that SC predictions of barrier heights consistently outperform their AP counterparts, while AP schemes are necessary in order to achieve accurate predictions of reaction energies for ozone reactions (Table 1). These corrections are especially important when ^1^O_2_ is a product, as SC errors can approach 20 kcal mol^–1^. These errors are so egregious that SC predictions for both ωB97X-V and ωB97M-V reactions energies are qualitatively incorrect in some cases, even predicting the wrong sign for the thermicity of Br^–^ ozone addition and then BrO_3_^–^ dissociation to BrO^–^ and ^1^O_2_.

The discrepancy between performance for *ΔE*_rxn_ and *ΔE*_TS_ is troubling, and we discuss the theoretical basis for this in Section S1.3. Here, we note simply that the best performance on *ΔE*_TS_ is achieved using spin-contaminated structures, where def2-QZVPPD RSMDs for ωB97X-V and ωB97M-V are 1.38 and 1.23 kcal mol^–1^, respectively. Both DFAs even predict energies within the bounds of chemical accuracy for about half SC transition structures. AP schemes are necessary to achieve tolerable predictions of *ΔE*_rxn_ for these functionals, resulting in RMSDs of 2.29 and 2.46 kcal mol^–1^ for reactions that do not produce ^1^O_2_. AP-ωB97M-V energies are particularly accurate for O_3_ scission reactions, and chemical accuracy is achieved in all cases. For ωB97X-V, AP errors are 1.48–2.58 kcal mol^–1^ in magnitude.

#### Wave Function Approaches

Recent developments in electronic structure theory have led to orbital optimization techniques that provide tractable alternatives to DFAs on the one hand and high-level CC or multireference approaches on the other. Orbital-optimized MP2 (OOMP2) has proven particularly promising, and regularization schemes like κ-OOMP2 have been shown to perform well across a variety of radical and closed shell systems.^107−109^ The strength of the regularizer κ is known to impact the quality of results.^108,109^ Benchmark predictions evaluated at various values of κ (Table S2) indicate κ = 1.45 provides the best results for the present systems, in line with a previous recommendation.^107^ With this method, only ^1^O_2_ exhibits symmetry breaking, so we only apply the AP scheme for reactions that produce this species. All other κ-OOMP2 values in Table 1 are uncorrected.

Using the def2-QZVPPD basis, this method yields RMSDs for *ΔE*_rxn_ and *ΔE*_TS_ of 5.12 and 2.10 kcal mol^–1^ relative to CCSDT(Q)/CBS results. In some cases, predictions for individual entries are nearly exact, while disagreement in the reaction energy for N(CH_3_)_3_ is in excess of 10 kcal mol^–1^. As for the DFAs, AP is necessary to achieve reasonable agreement for O_3_ scission reactions, but even here it exhibits errors in excess of 1 kcal mol^–1^.

#### Method Selection

Overall, the ωB97X-V and ωB97M-V DFAs perform surprisingly well for these systems, exceeding even the good expectations for hybrid density functionals established in previous work.^62^ Each of these methods outperforms κ-OOMP2, where a regularizer balancing the effects of static and dynamic correlation could not be achieved (Section S1.2). Based on these results, DFAs are used for the analysis that follows. The discrepancy between ωB97X-V and ωB97M-V performance is smaller than the overall errors of each of these methods relative to CCSDT(Q), such that the choice between these two methods is unlikely to make a material difference. On the basis of performance with the truncated def2-TZVPP basis set, which we use for systems below, we focus on results from ωB97X-V. The results in Table 1 lead us to expect this method to produce results accurate within 1-sigma errors of 1.25 kcal mol^–1^. Though not the primary purpose of this work, we emphasize here the importance of establishing an effective protocol for DFT modeling of ozone transition states and reaction energies, which are notoriously difficult problems in electronic structure theory.

### 3.2 EDA of Radical Ion Complexes

As documented in Section 1.1, a number of authors argue that explicit X → O_3_ one-electron transfer initiates ozonation chemistry for a wide variety of substrates X. We have used adiabatic energy decomposition analysis (EDA) to analyze this reactivity for 2M2B (**1**) and 4ADPA (**2**) as representative examples of alkenes/unsaturated polymers and PPDs, respectively.

**Figure 3:**
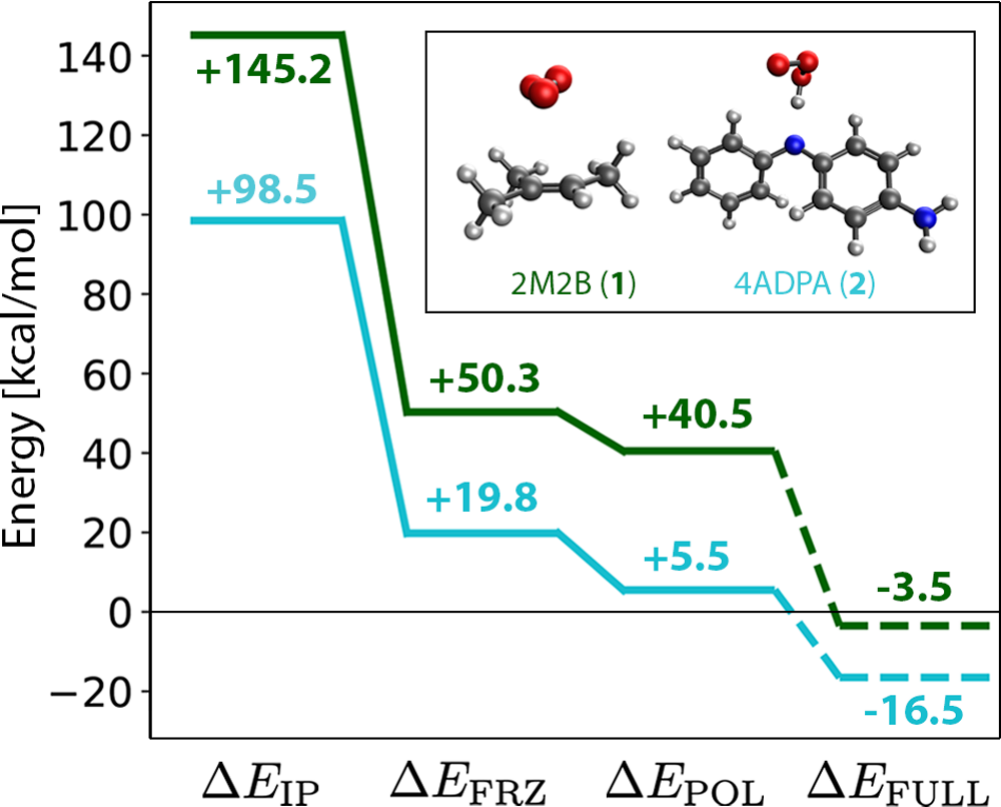
Energy decomposition analysis for CT complexes of 2M2B (**1**, top line) and 4ADPA (**2**, bottom line) with O_3_. Structural minima for biradical zwitterionic CT complexes could not be found on unconstrained surfaces, where spontaneous charge transfer (indicated by dashed lines) resulted in charge-neutral fragments and the structures shown in the inset. The zero of energy corresponds to the optimal geometries of isolated neutral fragments.

CT complexes between **1** and O_3_ on the series of ωB97X-V/def2-TZVPP EDA surfaces indicate that these structures are highly unstable relative to neutral fragments (Figure 3). The initial promotion to ionized fragments in this system is energetically expensive, with *ΔE*_IF_ = 145.2 kcal mol^–1^. This result is physically reasonable on the basis of experimental results^132^ for the first ionization energy of **1** and the electron affinity of ozone, 200.4 and 48.5 kcal mol^–1^, respectively, yielding *ΔE*_IF_^expt^ = 151.9 kcal mol^–1^. Interactions between these ionized fragments on the FRZ and POL surfaces are unable to compensate for the high promotion energy, yielding structures that are destabilized by 50.3 and 40.5 kcal mol^–1^, respectively, relative to the neutral fragments. Upon relaxation of all constraints, spontaneous charge transfer results in a neutral van der Waals (vdW) complex between **1** and O_3_ (Figure 3, inset). The inability of this approach to locate a structural minimum on the [**1**]^·+^[O_3_]^·–^ surface indicates that this complex cannot form spontaneously, and the vdW complex will preferentially form instead.

The situation is similar for **2**, which we take to be representative of other PPDs where these CT complexes have been repeatedly proposed.^73,74^ Ionization energies for PPDs are generally lower than those for olefins,^73^ and here we compute *ΔE*_IF_^comp^ = 98.5 kcal mol^–1^ for **2**. This lower energetic penalty results in CT complexes that are less energetically unfavorable than those for **1**, with *ΔE*_FRZ_ and *ΔE*_POL_ of 19.8 and 5.5 kcal mol^–1^, respectively (Figure 3). They are nonetheless still above the zero of energy corresponding to neutral fragments, and so here too relaxation of the CT constraint results in spontaneous electron transfer to afford neutral fragments. Upon subsequent geometry optimization cycles, the neutralized O_3_ molecule abstracts a proton from **2** as depicted in the inset of Figure 3. While the energy cost to form [**2**]^·+^[O_3_]^·–^ on the POL surface is relatively small, it is still unbound. Spontaneous reversion to neutral fragments upon relaxation of the constraints used to force formation of the CT complex indicates that such complexes do not form for these systems.

Taken together, the results of Figure 3 indicate that CT complexes like those described above do not form in the ozonation reactions of **1** and **2**. Inasmuch as these compounds are representative of olefins and PPDs more broadly, this conclusion carries over, and one-electron charge transfer should not be relied upon to explain the kinetics of ozone chemistry for olefins and PPDs, as is common (cf. Section 1.1).

### 3.3 Mechanisms of Natural Rubber Ozonation

**Figure 4:**
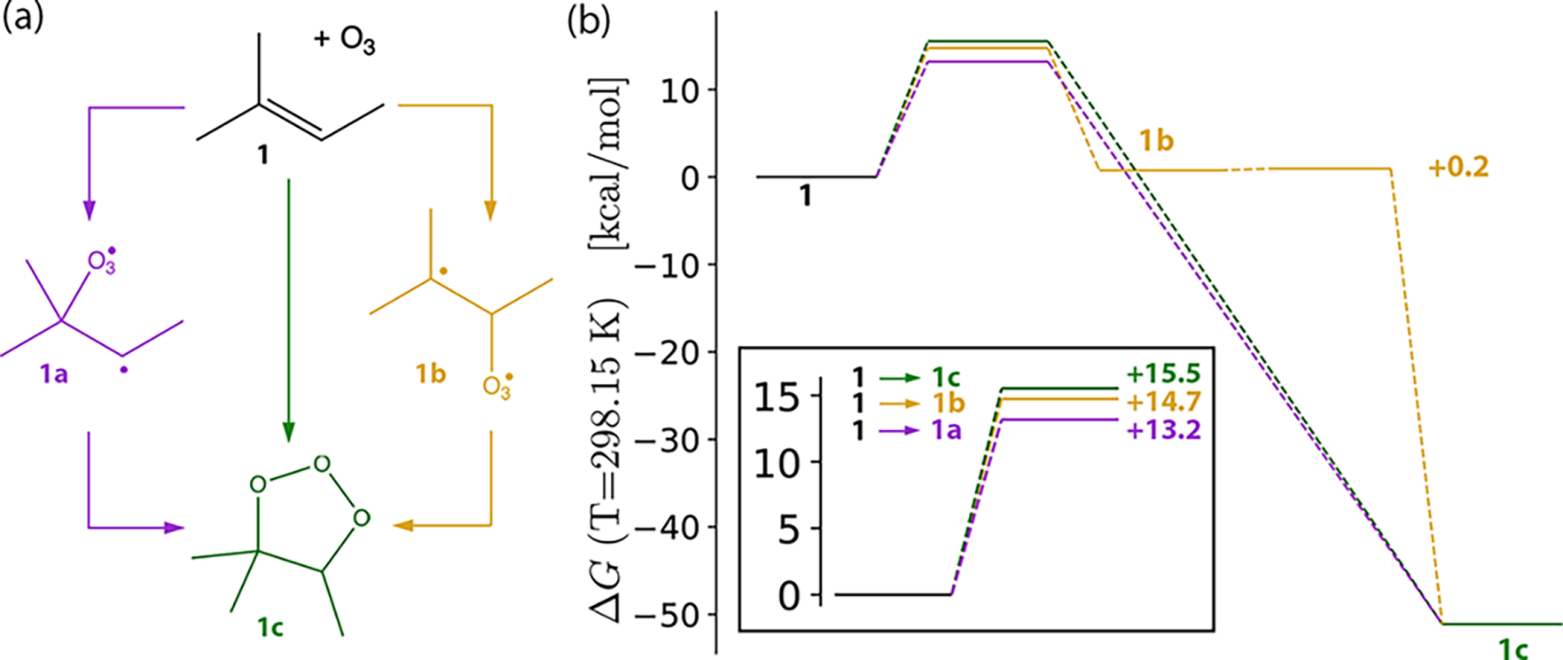
Mechanistic possibilities for the ozonation of 2-methyl-2-butene (**1**) to form primary ozonide **1c**. In addition to the symmetric addition of O_3_ to **1** (*ΔG*_298.15 K_^‡^ = 15.5 kcal mol^–1^) and the true DeMore pathway through **1b** (*ΔG*_298.15 K_^‡^ = 14.7 kcal mol^–1^), an asymmetric addition (*ΔG*_298.15 K_^‡^ = 13.2 kcal mol^–1^) *without* an isolable DeMore intermediate was identified.

Having eliminated the possibility of a CT mechanism for **1**, two possibilities remain: concerted (Criegee) and stepwise (DeMore) addition of O_3_ across the **1** double bond. Conceptually, two distinct DeMore pathways exist for **1**, corresponding to initial reactions forming secondary (**1a**) and tertiary (**1b**) carbon radicals (Figure 4[a]). Each of these structures may then collapse to the primary ozonide (**1c**). Therefore, we expect at least three distinct transition structures on the **1**–O_3_ PES.

The Criegee mechanism for addition of O_3_ to **1**, where the symmetric formation of two C–O bonds was unambiguously confirmed with intrinsic reaction coordinate (IRC) analysis,^123,124^ possesses a barrier height of 15.5 kcal mol^–1^ on the ωB97X-V/def2-TZVPP surface (Figure 4[b]). Analysis of the DeMore structures **1a** and **1b** was more complicated. While a structure corresponding to **1a** with a single imaginary frequency corresponding to the formation of the C2–O_3_ bond was identified, IRC on this structure identified it as a saddle point connecting the vdW complex and primary ozonide **1c**. Hence, this structure actually represents an asymmetric pseudo-Criegee structure, with a barrier of 13.2 kcal mol^–1^. A similar structure that initially forms the C3–O_3_ bond was also identified. These structures are DeMore-like in the asymmetric formation of C–O bonds, but they do not result in true DeMore intermediates, i.e. local minima on the ωB97X-V/def2-TZVPP surface. Nevertheless, asymmetric addition to the double bond in **1** is more favorable than symmetric addition by approximately 2.3 kcal mol^–1^ at the ωB97X-V/def2-TZVPP level.

In addition to these three transition structures, a true DeMore pathway exists for the tertiary radical intermediate **1b**, where IRC analysis does identify a local minimum for this species. The ωB97X-V/def2-TZVPP barrier height for **1** → **1b** is 14.7 kcal mol^–1^ (Figure 4[b]), meaning this will be a minor pathway relative to the pseudo-Criegee structures reported above. A second transition structure (*ΔG*^‡^ = 0.2 kcal mol^–1^) was identified for the collapse of **1b** → **1c**. It is possible that solvation effects could stabilize this pathway, as has been observed in other systems,^54^ but we do not explore this here as it is irrelevant to the rubber system of interest. Though the differences between these barrier heights are on the order of the tolerance of our ωB97X-V model (Section 3.1 and Table 1) and we have omitted any effects of the tire matrix, these values provide an estimate of the degree of O_3_ reactivity necessary to protect rubber in tires.

Overall, the lowest energy pathway for **1** ozonation is an asymmetric Criegee addition, which possesses a barrier height of a mere 13.2 kcal mol^–1^ at the ωB97X-V/def2-TZVPP level. This and other small barrier heights for this system underscore one difficulty of developing rubber antiozonants. Candidate molecules must possess even smaller transition structure energies in order to effectively scavenge O_3_. As seen below, PPDs are among the few molecules that achieve this threshold of reactivity.

### 3.4 Mechanistic Pathways for PPDs

PPDs are highly reactive substrates in oxygen and ozone chemistry, and a number of potential pathways for the reaction of ozone with these compounds can be imagined.^41,42^ This panoply of reaction intermediates is reminiscent of the ozone chemistry of aniline^67^ and phenolic systems,^64^ complicating efforts to identify the mechanism of quinone formation. While a few overarching frameworks have been suggested,^12,41^ no stepwise mechanisms for 6PPD quinone formation have been proposed. We begin the analysis of PPD ozonation with discussion of the kinetics and thermodynamics of ozonation steps for 4ADPA (**2**) in Section 3.4.1, demonstrating that the particularly high activity of PPDs toward O_3_ stems from direct interactions with the PPD ring system. We then consider the effects of N-alkylation on the kinetics of key ozonation steps in Section 3.4.2. Finally, we discuss proposals that PPD quinones form through quinone diimines (QDIs) in Section 3.4.3.

#### 3.4.1 Ozonation of 4ADPA

**Figure 5:**
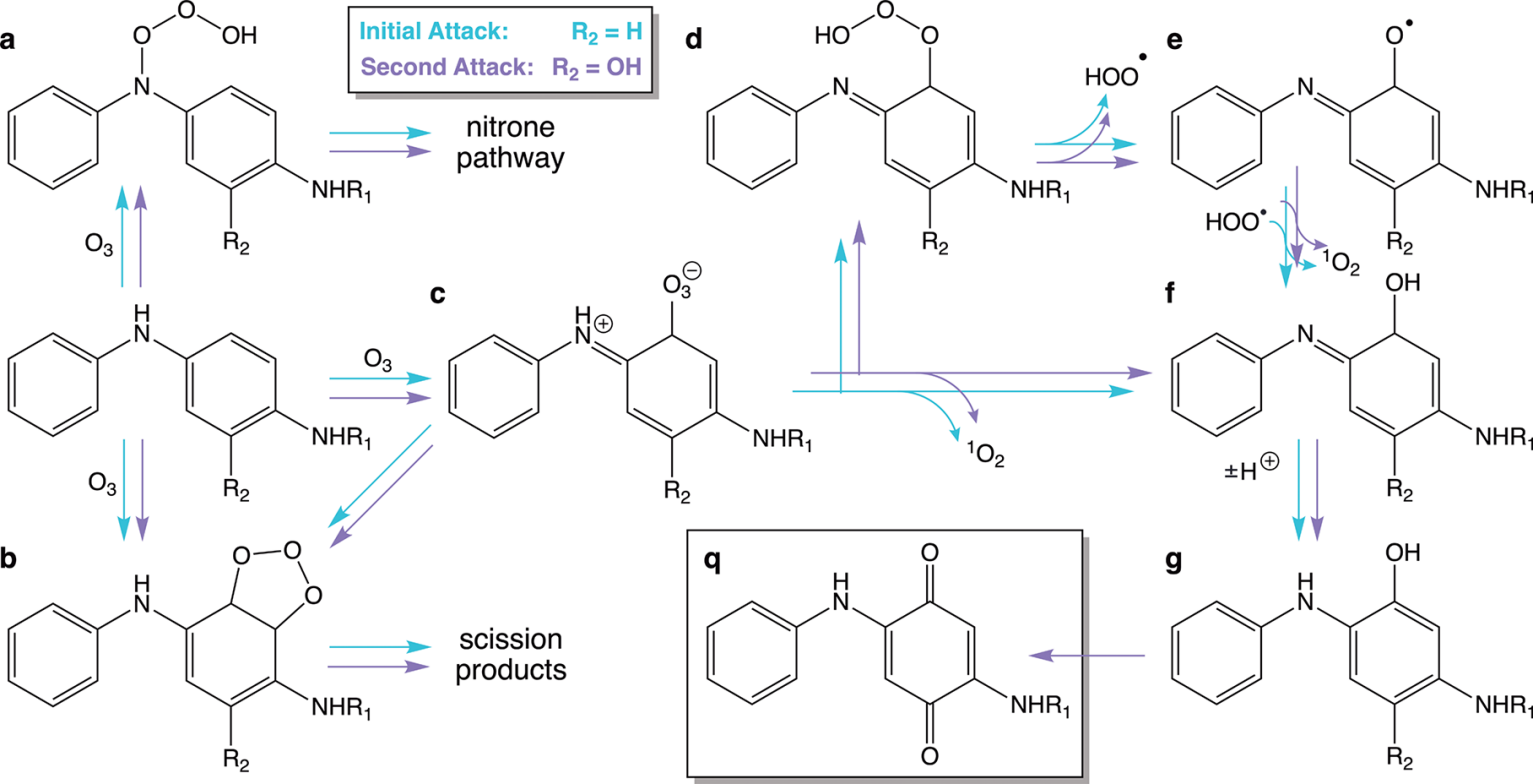
Selected reaction pathways for PPDs and ozone. In general, each structure represents a collection of regioisomers. Two sequential additions of ozone to the PPD ring system can afford hydroquinone **g** (*R*_2_ = OH), which is readily oxidized to quinone **q**. *R*_1_ = H, Me, and 1,3-dimethylbutyl correspond to 4-ADPA (**2**), *N*-Me-PPD (**3**), and 6PPD (**4**), respectively.

Charge transfer mechanisms aside (Section 3.2), we consider a number of initial steps for the reactivity between 4-ADPA (**2**) and O_3_, including linear addition of O_3_ to N atoms, insertion of O_3_ into N–H bonds, and both Criegee (concerted) and Demore (stepwise) addition to the aromatic ring (Figure 5). In all cases, a number of regioisomers and conformers were considered. While energetic data for each of these can be found in the SI, here we present transformation energies for only the most stable conformers and select other structures of interest.

**Figure 6:**
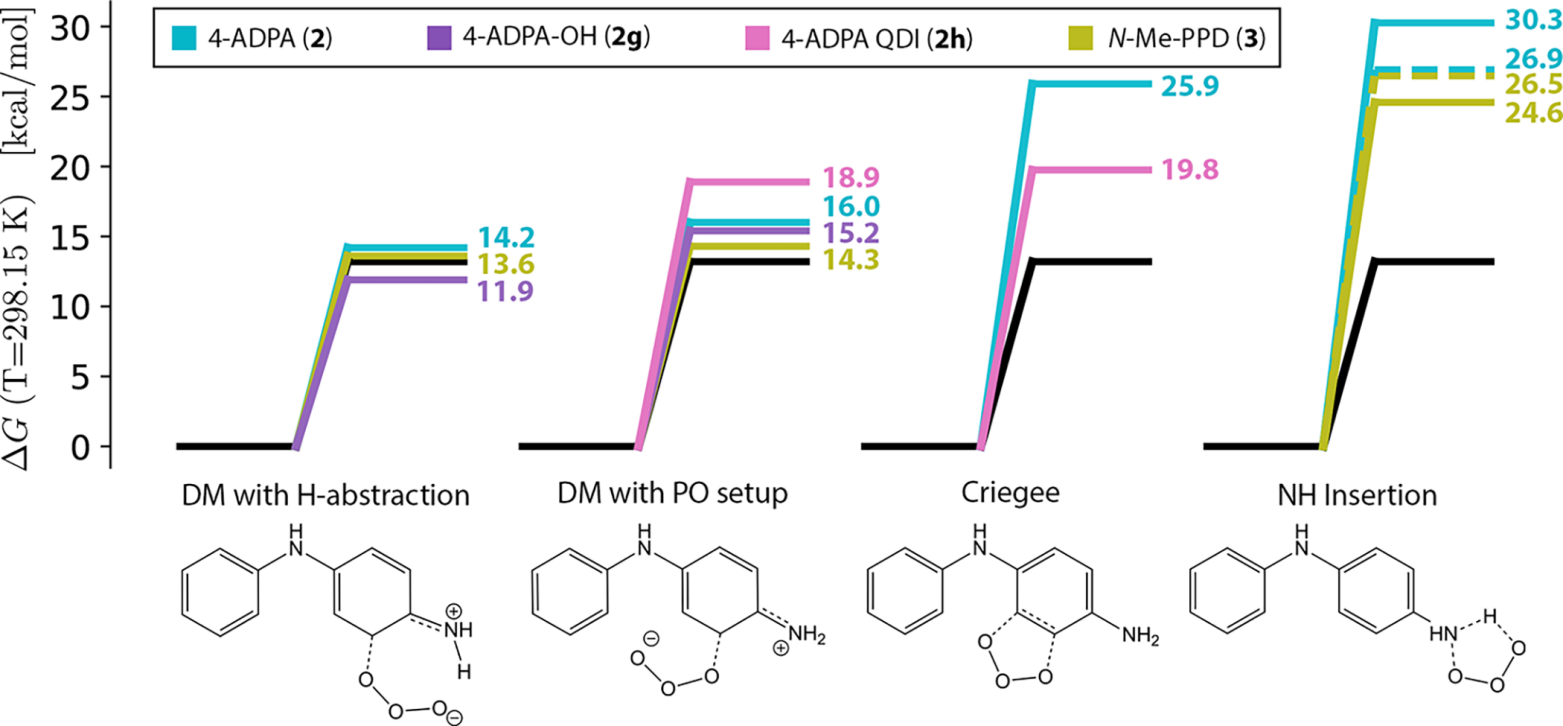
Free energy barrier heights (*T* = 298.15 K) for PPD ozonation pathways compared to the minimum barrier pathway for 2M2B ozonation (**1**, solid black lines). Transition structures are shown for 4-ADPA transformations. For N–H insertion reactions, solid and dashed lines correspond to insertion at terminal and central N atoms, respectively. DeMore (DM) additions of O_3_ to the rings of 4-ADPA (**2**), 4-ADPA–OH (**2g**), and MePPD (**3**) are the only transformations that are kinetically competitive with **1** ozonation. DeMore TSs with secondary H-abstraction interactions show additional stabilization.

Of considered steps, direct interaction between PPD amines and O_3_ corresponds to the least favorable transition structures, contrary to historical and prevailing opinion (Figure 6).^37,40,72−74^ Insertions into the N–H bond exhibited lower barrier heights than N additions, but even here *ΔG*_298.15 K_^‡^ = 30.3 and 26.9 kcal mol^–1^ for terminal and central amines **2a**, respectively (Figures 5 and 6). Such high barriers are qualitatively consistent with the high value of the benchmark quality result for NH_3_ insertion (*ΔE_^‡^_*_0 K_ = 23.1 kcal mol^–1^) from Trogolo et al.^62^ Experimental evidence also supports the conclusion that N–O bond formation is not the major path of ozone consumption in PPDs. Rate constants for PPD ozonation in CCl_4_ are generally on the order of 5–20 × 10^6^ M^–1^ s,^74,133^ while those for aliphatic amines (where only the N–O addition/N–H insertion pathways exist) in aqueous solution increase from 9.3 × 10^4^ to 4.1 × 10^6^ M^–1^ s^–1^ with alkyl substitution.^59,80,134^ The influence of solvent can be roughly determined by reference to results for triethylamine (the upper bound in this range) in CCl_4_, where the rate constant is 2.3 × 10^4^ M^–1^ s^–1^.^133^ If the primary pathway of PPD ozonation involved N–O interaction, we would expect a rate constant below this final value. That ozonation rate constants for PPDs are at least 2 orders of magnitude faster indicates the presence of more reactive ozonation channels.

Instead, direct interaction with the PPD ring results in more stable—thereby, more accessible—transition structures. Among these interactions, concerted addition of ozone to the ring system, which has been proposed for other electron-rich aromatics,^68,69^ to form primary ozonide **2b** is the least favorable. The barrier for the minimum energy Criegee transition structure (*ΔG*_298.15 K_^‡^ = 25.9 kcal mol^–1^) is on the same order as that for N–H insertion. However, asymmetric addition to the ring system is significantly more accessible, with barrier heights ranging from 14.2–19.0 kcal mol^–1^. Conformational details of these transition and product structures explain the range of *ΔG*^‡^ values in these systems and provide significant insight into the reactivity of PPDs and likely fate of these structures.

Each of the most stable DeMore transition structures in **2** is stabilized by a secondary interaction between the substrate and O_3_. This additional stabilization comes either from a second C–O interaction or interaction between the O_3_ terminus and the amine hydrogens. These latter conformations are particularly stable, and the lowest energy structure exhibits a barrier height of *ΔG*_298.15 K_^‡^ = 14.2 kcal mol^–1^, on par with the minimum barrier for **1** within the uncertainty of our ωB97X-V/def2-TZVPP treatment (see Table 1). IRC computations indicate a single C–O bond formation, but the resulting intermediate is primed for amine H abstraction, a reaction that affords the hydroperoxy imine, **2d**. In both types of DeMore transition structures—those set up for primary ozonide formation and for H abstraction—4ADPA ozonation pathways initiated through interaction with the C atoms of the PPD ring are significantly more accessible than interactions with PPD amines.

These transition structures indicate that transformations from various isomers of DeMore adduct **2c** to primary ozonide **2b** and hydroperoxy-imine **2d** represent the most significant early stage intermediates of **2** ozonation. As a result, the reactivity of these compounds determines the distribution of the products for the overall reaction between **2** and ozone. 1,2-Primary ozonide **2b** is expected to decompose into open-chain scission products^64,67,69^ similar to the wide variety of experimentally determined PPD ozonation products.^42^ While not the focus of the present study, the wax-like structure of these products (especially in the 6PPD system, which has a longer alkyl tail) makes them potential candidates for the film formation mechanism of PPDs in tires.^37^ For present purposes, the opening of the PPD ring is the most salient feature of these products, making them poor candidates for the formation of **2** quinone, and we do not consider them further.

**Figure 7:**
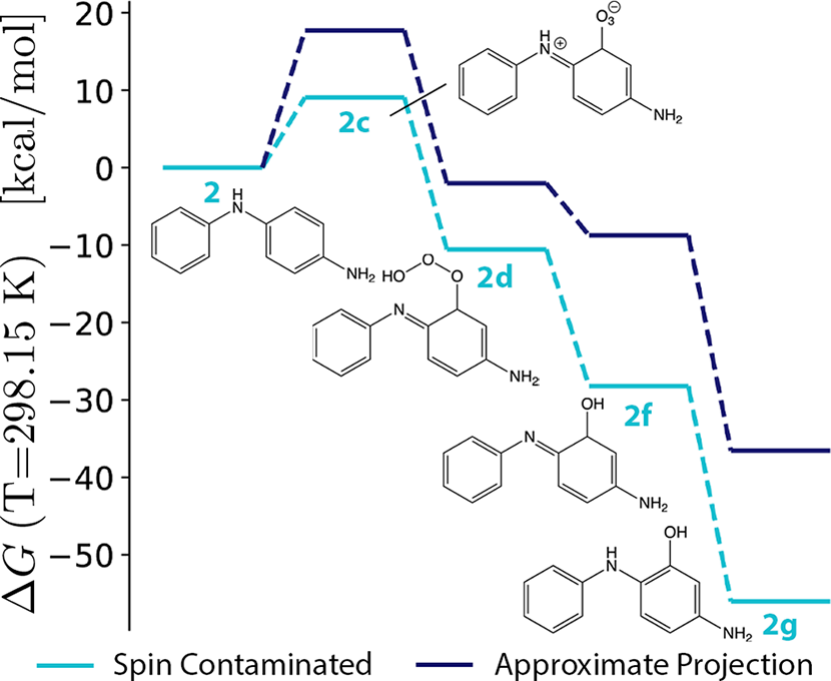
Reaction energies (*ΔG*_rxn,298.15 K_) for steps from 4ADPA (**2**) to 4ADPA–OH (**2g**) at the SC- and AP-ωB97X-V/def2-TZVPP levels. Following the initial ozonation step to form **2c**, both methods indicate a strongly exothermic reaction cascade.

Formation of **2d**, on the other hand, is a promising step toward the quinone, forming a necessary C–O bond and preserving the ring structure. This species is primed for the loss of hydroperoxy radical (HOO^•^) to form alkoxy radical **2e**,^67,69,70^ which may abstract H^•^ from HOO^•^ producing ^1^O_2_. This reaction affords hydroxyl imine **2f**, which may rearomatize to form hydroxyl-4ADPA (**2g**) with catalytic amounts of H^+^.^67^ While potentially a minor pathway, it is also possible that alkoxy radical **2e** immediately recombines with HOO^•^ and loses H_2_O to promptly form the quinone **2q**, analogous to aqueous systems.^71^ Due to the difficulty of identifying transition structures, we do not report barrier heights for these transformations. Still, overall reaction energies (Table 2 and Figure 7) indicate that each individual step is thermodynamically feasible. The citations for each step also provide literature precedent for each of these transformations in similar systems, though other pathways from **2d** to **2g** can be imagined.^66,68,69^

**Table 2:** Barrier Heights and Reaction Free Energies (T = 298.15 K) for PPD Ozonation on Both SC- and AP-ωB97X-V/def2-TZVPP Surfaces^a^

	SC		AP	
reaction	*ΔG*^‡^	*ΔG*_rxn_	*ΔG*^†^	*ΔG*_rxn_
**1** → **1c**	13.2	–51.1	15.4	–42.5
**2** → **2a**	26.9	–8.0	38.4	0.7
**2** → **2b**	25.9	–16.5	34.6	–7.9
**2** → **2c**	14.2	9.06	19.5	17.7
**2c** → **2d**	–	–19.6	–	–19.7
**2d** → **2f**	–	–17.7	–	–6.7
**2f** → **2g**	–	–27.8	–	N/A
**2g** → **2c**′	11.9	8.3	17.1	14.9
**3** → **3a**	24.6	–10.3	14.0	–1.7
**3** → **3c**	13.6	8.6	18.8	16.0
**2h** → **2i**	19.8	–36.0	28.4	–27.4
**2h** → **2j**	18.9	3.4	20.7	12.4

a SC energies are more accurate for *ΔG*^‡^, while AP energies are more accurate for *ΔG*_rxn_ (Section 3.1). Results for the lowest energy 2M2B (**1**) ozonation pathway are included for reference. [Units: kcal mol^–1^].

Regardless of the particular steps, production of hydroxyl-4ADPA **2g** through direct **2**–O_3_ interaction is the salient feature of this mechanistic proposal. This motif is analogous to ozonation of anilines,^67^ catechols,^66^ and pyrazoles,^70^ where hydroxylated aromatics are significant products. Furthermore, recent experimental work identified the presence of 6PPD’s analogue of **2g** in snow samples collected along major roadways.^42^ These hydroxyl intermediates aid the function of PPDs in tires, as these derivatives possess similar reactivity toward O_3_ as their parent compounds.^60^ Indeed, addition of ozone to **2g** to form hydroxylated DeMore intermediate **2c**′ proceeds with a minimum barrier height of 11.9 kcal mol^–1^, lower than the minimum barrier for both **1** and **2**. Because **2g** is even more reactive toward O_3_ than **2**, an initial ozonation of **2** to form **2g** “commits” the substrate to a second ozonation. Each of the reaction pathways available to **2c** exists for **2c**′ (where the prime indicates a hydroxylated species), which may decompose into scission products through primary ozonide **2b**′ or form hydroquinone **2g**′ through **2d**′ (Figure 5). This hydroquinone can slowly oxidize to 4ADPA quinone **2q** at ambient conditions^135^ or more rapidly through further reaction with O_3_.^68,71^ Though not shown in Figure 5, adduct **2c**′ may alternatively abstract the *ipso* H from the ring to form the quinone, similar to the mechansim proposed for phenolates.^68^

Overall, this cascade of reactions results in a molecular mechanism for the formation of 4ADPA quinone (**2q**) from **2** through a series of kinetically and thermodynamically favorable transformations. While the reactions in Figure 5 do not account for all of the experimentally determined products of PPD ozonation^42^ and are not intended to be exhaustive, they demonstrate a viable path to the quinone that is linked to the high activity of PPDs toward O_3_. The initial nucleophilic attack of O_3_ by the PPD ring has not been previously proposed but critically occurs with a barrier height close to that for 2M2B ozonation (Section 3.3). Additionally, consumption of two molar equivalents of O_3_ is consistent with experimental results.^18^ Thus, this channel allows kinetic scavenging of ozone, even for aryl PPD **2**.

#### 3.4.2 Effects of N-Alkylation

It is well-known that alkyl-aryl PPDs (like 6PPD) are more reactive toward ozone than aryl PPDs like our surrogate compound **2**.^3,4,35^ Still, inclusion of the 6PPD alkyl chain into our model system would result in a nearly 50% increase in the size of our system as well as introducing a significant degree of conformational flexibility. We therefore model the kinetic effects of alkylation using *N*-Me-PPD (**3**), which is more computationally tractable and should capture the lion’s share of the relevant effects.

Many authors attribute increased ozonation rates to more stable interactions with the nitrogen atom,^35^ but we have already noted that these are not significant reaction channels for **2**. N–H insertion pathways remain uncompetitive in **3**. Unsurprisingly, alkylation of the terminal amine does not affect that barrier height for insertion into the central N–H bond significantly, and we have *ΔG*_298.15 K_^‡^ = 26.5 kcal mol^–1^ for this reaction. Methylation of the terminal nitrogen does increase the reactivity of its N–H bond considerably, resulting in an insertion barrier of 24.6 kcal mol^–1^ (cf. the barrier of 30.3 kcal mol^–1^ in **2**), but this is still relatively large.

**Figure 8:**
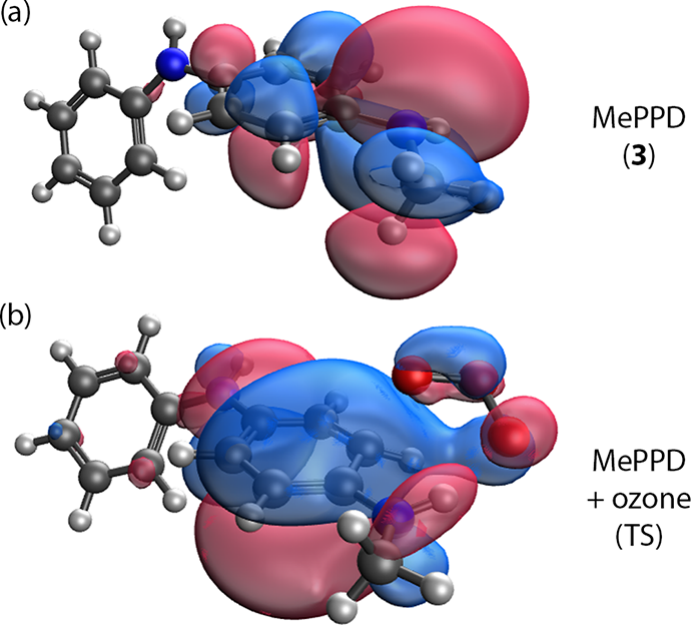
Intrinsic bonding orbitals (IBOs) for (a) MePPD (**3**), showing delocalization of the N lone pair stabilized by methyl hyperconjugation and (b) DeMore-like TS for **3** with a secondary interaction between the amine H and O_3_.

Instead, the experimental effects of alkylation can be attributed to additional stability in the interactions between ozone and the ring system, mediated through the N atom’s connection to the π-system. The DeMore-plus-H-abstraction transition structures that lead to hydroperoxy imine **3d** at the methylated amine are again particularly stable, with a minimum barrier height of *ΔG*_298.15 K_^‡^ = 13.6 kcal mol^–1^ relative to isolated fragments. This stabilization can be understood with reference to the “lone pair” (LP) orbital on the terminal N atom in **3** (Figure 8[a]). Hyperconjugation of the methyl C–H density supports interactions through the ring π-system in **3**, resulting in significant delocalization of the amine LP through the ring. In the transition structure for O_3_ addition (Figure 8[b]), this orbital clearly exhibits the nascent interaction between the floating O_3_ and the amine H, even though IRC confirms the single C3–O bond formation. Even for **3**, the minimum predicted barrier height of 13.6 kcal mol^–1^ is larger than that for **1** (*ΔG*_298.15 K_^‡^ = 13.2 kcal mol^–1^), although this difference is well within the 1.25 kcal mol^–1^ 1-sigma error of our method (Table 1). In any case, these results underscore how difficult it is to identify molecules with lower O_3_ barrier heights than **1** and related compounds.

The role of amine H atoms in achieving this high degree of ozone reactivity has gone unrecognized in previous work on PPD systems. While barrier heights for certain other DeMore structures (like the precursors to 1,2-primary ozonides) are competitive, stabilization from H-abstraction leads to particularly active reaction channels. As seen above (Section 3.4.1), these channels open a path to quinone formation, and there is an inextricable link between the activity of 6PPD as an antiozonant and its toxicity through the quinone.

#### 3.4.3 Pathways through the Quinone Diimine

While the preceding discussion provides strong evidence for one pathway to PPD quinones, alternative mechanisms are present in the literature. Indeed, recent experimental evidence has been interpreted to suggest quinone formation proceeds through the 6PPD quinone diimine (QDI).^41^ Specifically, mass spectrometry of the ozonation products of 6PPD results in a peak with an integrated mass of 283.1798 Da, which the authors attributed to the [M + H]^+^ ion of 6PPD QDI–OH. This interpretation is supported by a body of work detailing the *in situ* formation of 6PPD QDI itself,^22,23^ and others have proposed similar chemistry.^42^ We evaluate the possibility of 6PPD QDI ozonation to form 6PPDQ through the QDI with reference to the model 4-ADPA QDI (**2h**), assuming once again this system captures the fundamental chemistry.

**Figure 9:**
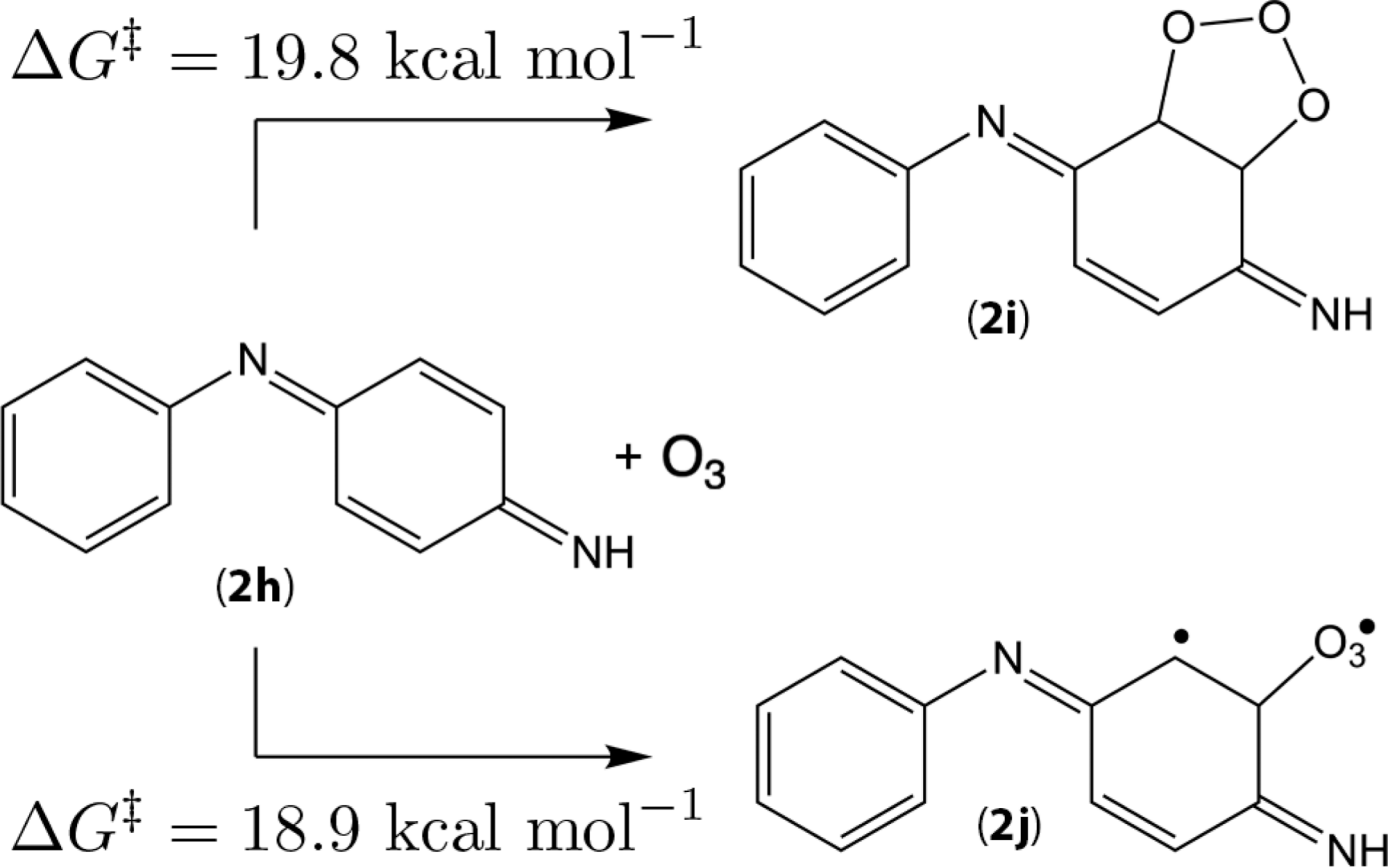
Reaction pathways for ring ozonation in 4ADPA QDI (**2h**). Neither primary ozonide **2i** nor DeMore intermediate **2j** is likely to result in products conducive to quinone formation.

Analogous to both **2** and **3** as discussed in Sections 3.4.1 and 3.4.2 above, **2h** possesses transition structures corresponding to both Criegee (concerted) and DeMore-like (stepwise) addition of O_3_ to the QDI ring system (Figure 9). These two pathways are similarly facile in the QDI system. Quantitatively, the minimum barrier for concerted and stepwise addition to **2h** is 19.8 kcal mol^–1^ and 18.9 kcal mol^–1^, respectively. It is noteworthy that the absence of amine H atoms in **2h** corresponds to the absence of extremely low barrier ozonation pathways as were seen for **2** and **3**, underscoring the importance of H-abstraction for PPD ozonation. Instead, in the case of **2h** DeMore adducts, the most stable DeMore transitions are accompanied instead by secondary interactions with an additional C atom in the PPD ring. Hence, as far as ring interactions with **2h** are concerned, the significant pathways will progress through primary ozonides, ultimately resulting in scission products. In particular, it is difficult to see how these structures could produce quinones.

It therefore seems more likely that the experimentally determined^41^ 6PPD QDI–OH forms through the oxidation of 6PPD–OH (**4g**) to the QDI, rather than the hydroxylation of the QDI. These considerations also indicate that the QDI is unlikely to be a precursor to the quinone. All of the results of the present study suggest, instead, that a direct attack of the 6PPD ring initiating the pathway to the quinone occurs through 6PPD–OH, which should exhibit increased reactivity toward ozone than even the parent 6PPD.

### 3.5 Implications for Nontoxic PPD Design

The preceding mechanistic work highlights the vulnerability of rubber to degradation by ozone and explains the distinct ability of PPDs to afford protection against this degradation. As identified through analysis of key, rate-determining steps in the mechanism of PPD ozonation, critical features of this chemistry that result in the formation of highly toxic 6PPD quinone should inform ongoing work to identify replacements for 6PPD.

The mechanistic work in this report has dispelled a number of reports in the literature that suggest the ozonation of both alkene and PPD systems is initialized by one-electron transfer from the subtrate to ozone.^76−79^ This provides theoretical backing to the perhaps obvious conclusion that predicting ozonation capacity is more complicated than determining ionization potentials, even if this parameter is relatively predictive within a single class of molecules.^74^ Instead, the subtlety of the mechanistic results above indicate that atomistic details of reactivity must be taken into account.

Under the previous paradigm that ozonation of PPDs is facilitated through their N atoms,^37,40,72^ prevention of quinone formation through deactivation of the aromatic ring system is a logical approach to reduce the toxicity of 6PPD. The results above, however, demonstrate direct attack by the aromatic ring is significantly more favorable, suggesting that such approaches will be unsuccessful, as they will poison the antiozonant capacity of 6PPD. The discovery of the importance of secondary interaction with amine H atoms for stabilizing transition structures suggests the necessity of these atoms also has bearing on PPD design. Specifically, while compounds that lack the potential for these reactions may still exhibit reactivity toward O_3_ (e.g., 6PPD QDI^42^), these reactions are unlikely to afford kinetic protection against rubber ozonation.

Present results indicate that activation of the PPD ring significantly increases activity toward O_3_ (Figure 6). This is seen first in the minor increase in reactivity upon *N*-alkylation, which has been known for some time,^3,4,35^ and then by the significant increase upon hydroxylation. Though ring activation improves antiozonant performance, it also facilitates the reactions that can ultimately produce PPD quinones. This is perhaps the most important result of this study, in that it provides a direct, atomistic link between the function of 6PPD as an antiozonant and its aquatic toxicity through the quinone.^12−17^ While this suggests an obvious difficulty in continued use of the PPDs on the market today, all is not lost. It may be possible to trap intermediates in the reaction prior to formation of the (hydro)quinone, but the scheme above indicates that this will likely reduce the molar equivalents of O_3_ consumed by the PPD. Alternatively, the mechanistic insights of the present work explain both the rapid reactivity of PPDs toward O_3_ and demonstrate the channel of formation for the toxic quinone. These provide principles for rational design of effective antiozonants that inform ongoing efforts to develop nontoxic alternatives to 6PPD in tires.

## Supporting Information Available

S1: Further analysis of computational models; S2: Conformer specifications; Figure S1: Definition of conformer labeling for PPD DeMore adducts; Figure S2: Definition of conformer labeling for PPD-OH DeMore adducts; Figure S3: Definition of conformer labeling for PPD primary ozonides; Figure S4: Definition of conformer labeling for hydroxylated PPDs; Table S1: Errors in spin-contaminated (SC) and approximate projection (AP) singlepoint energies for benchmark van der Waals (vdW) complexation energies; and Table S2: Effect of regularizer strength (κ) on performance of κ-OOMP2 for benchmark ozonation reactions (PDF)
Absolute energies for determining transition and reaction energies (XLSX)
Stationary point geometreis (ZIP)
