# Supplementary material for: Computational Studies of Rubber Ozonation Explain the Effectiveness of 6PPD as an Antidegradant and the Mechanism of Its Quinone Formation
Source: Environ Sci Technol. 2023 Mar 24;57(13):5216–30. doi: 10.1021/acs.est.2c08717 (PMC10079164; doi:10.1021/acs.est.2c08717)
Supplement: Supplementary file 1 — es2c08717_si_001.pdf [file es2c08717_si_001.pdf]

Supporting Information: Computational studies of rubber ozonation explain the effectiveness of 6PPD as an antidegradant and the mechanism of its quinone formation

Elliot Rossomme,[*,†,‡,¶,§] William M. Hart–Cooper,[†] William J. Orts,[†] Colleen M. McMahan,[*,†] and Martin Head–Gordon[*,‖,¶,§]

†*Bioproducts Research Unit, Agricultural Research Service, U.S. Department of Agriculture, Albany, CA 94710, United States*

‡*Berkeley Center for Green Chemistry, University of California, Berkeley, CA 94720, United States*

¶*Chemical Sciences Division, Lawrence Berkeley National Laboratory, Berkeley, California 94720, United States*

§*Department of Chemistry, University of California, Berkeley, CA 94720, United States*

‖*Kenneth S. Pitzer Center for Theoretical Chemistry, University of California, Berkeley, CA 94720, United States*

E-mail: elliot.rossomme@gmail.com; colleen.mcmahan@usda.gov; mhg@cchem.berkeley.edu

Contents

List of Figures **S3**

List of Tables **S3**

S1 Further analysis of computational models **S4**

S1.1 Benchmark values for vdW complexes S4

S1.2 The effect of regularizer strength in κ-OOMP2 . . . . S5

S1.3 Understanding spin contamination in DFA energies S7

S2 Conformer specifications **S8**

References **S12**

List of Figures

S1 Definition of conformer labeling for PPD DeMore adducts. R = H (4ADPA), Me (MePPD). S9
S2 Definition of conformer labeling for PPD-OH DeMore adducts. R = H (4ADPA), Me (MePPD). S10
S3 Definition of conformer labeling for PPD primary ozonides. R = H (4ADPA), Me (MePPD). S11
S4 Definition of conformer labeling for hydroxylated PPDs. R = H (4ADPA), Me (MePPD). S11

List of Tables

S1 Errors in spin-contaminated (SC) and approximate projection (AP) single-point energies for benchmark van der Waals (vdW) complexation energies. For κ-OOMP2 (κ = 1.45) results, no spin polarization was observed for O_3 or any vdW complex, so "SC" does not apply and AP corrections are identically zero. Reference values are CCSDT(Q) results extrapolated to the CBS limit obtained from Ref. 1. Highlighted boxes represent the best performing methodology for a given parameter at a given basis set truncation. S5
S2 Effect of regularizer strength (κ) on performance of κ-OOMP2 for benchmark ozonation reactions. Empty columns for approximate projection (AP) results indicate an absence of spin polarization for all relevant species and the given method. S6

S1 Further analysis of computational models

Spin projection. Yamaguchi's approximate spin projection (AP) method[2] was used to address spin contamination in both DFA and κ-OOMP2 computations. Procedurally, the degree of spin contamination in the singlet reference state is represented by the parameter

$$\alpha = \frac{^1\langle \hat{\mathbf{S}}^2 \rangle_{\mathrm{SC}}}{^3\langle \hat{\mathbf{S}}^2 \rangle - {}^1\langle \hat{\mathbf{S}}^2 \rangle_{\mathrm{SC}}}, \tag{1}$$

where $^1\langle \hat{\mathbf{S}}^2 \rangle_{\mathrm{SC}}$ and $^3\langle \hat{\mathbf{S}}^2 \rangle$ are the expectation values of the total spin operator for the spin-contaminated (SC) singlet reference state and a high-spin triplet state, respectively. This parameter is then used to project energetic contributions from the high-spin state out of the targeted singlet reference, affording the corrected energy

$$^1E_{\mathrm{AP}} = {}^1E_{\mathrm{SC}} + \alpha \left(^1E_{\mathrm{SC}} - {}^3E \right) \tag{2}$$

using the energies of the triplet and spin-contaminated singlet reference states, ^3E and $^1E_{\mathrm{SC}}$. Both SC and AP energies are reported for all reactive oxygen species, including ozone, singlet oxygen, and various intermediates and transition states that were determined to resemble these molecules on the basis of the values of $\langle \hat{\mathbf{S}}^2 \rangle$ for computed reference states.

S1.1 Benchmark values for vdW complexes

In contrast to transition structures and reaction energies, both spin contaminated and approximate projection (AP)[2] schemes yield adequate results for van der Waals (vdW) complexation energies (ΔE_{vdW}) with O_3 (Table S1). Reference values obtained from Ref. 1 were computed using CCSDT(Q) and extrapolated to the complete basis set limit. Results using the ωB97X-V[3] and ωB97M-V[4] density functionals, as well as the κ-OOMP2 method (κ = 1.45),[5] are within chemical accuracy for almost all species. Furthermore, while not employed here for the sake of simplicity, we expect even better agreement could be achieved

Table S1: Errors in spin-contaminated (SC) and approximate projection (AP) single-point energies for benchmark van der Waals (vdW) complexation energies. For κ-OOMP2 ($\kappa = 1.45$) results, no spin polarization was observed for O_3 or any vdW complex, so "SC" does not apply and AP corrections are identically zero. Reference values are CCSDT(Q) results extrapolated to the CBS limit obtained from Ref. 1. Highlighted boxes represent the best performing methodology for a given parameter at a given basis set truncation.

	Error in ΔE (kcal mol^{-1})										ΔE_{ref}
	def2-TZVPP					def2-QZVPPD					CBS
	ωB97X-V		ωB97M-V		κ-OOMP2	ωB97X-V		ωB97M-V		κ-OOMP2	CCSDT(Q)
Substrate	SC	AP	SC	AP	–	SC	AP	SC	AP	–	
vdW Complexation Energies											
C_2H_4	-0.05	-0.76	-0.10	0.40	-0.40	0.23	-0.38	0.20	-0.12	-0.71	-2.01
C_2H_2	-0.10	-0.70	-0.13	0.34	-0.38	0.15	-0.36	0.13	-0.11	-0.61	-1.83
HCN	-0.12	-0.04	-0.09	0.09	-0.32	0.10	0.31	0.16	0.33	-0.57	-1.96
HCl	-0.10	-0.06	-0.10	0.05	-0.37	0.28	0.45	0.33	0.50	-0.26	-2.23
NH_3	-0.32	-0.12	-0.38	0.04	-0.40	0.20	0.52	0.19	0.53	-0.40	-2.75
$N(CH_3)_3$	-0.27	-0.91	-0.38	0.59	-1.01	0.31	-0.20	0.27	0.07	-1.16	-3.80
Br^-	-3.34	-1.33	-4.09	2.75	0.41	-1.80	0.45	-2.28	-0.80	0.07	-6.13
RMSD	1.27	0.72	0.96	1.56	0.52	0.71	0.39	0.89	0.43	0.63	–

with use of counterpoise corrections (CPCs) to correct for the basis set superposition error (BSSE) that arises in these computations.[6] When the def2-TZVPP basis set[7] is used, the errors for the Br^- complex are significantly larger than other species, reflecting the necessity of diffuse orbitals in modeling anions. Significantly improved performance is achieved with the def2-QZVPPD set.[8] While values of ΔE_{vdW} do not play a role in the main work of this study, these results provide additional insight into discrepancies in the performance of spin-contaminated (SC) and approximately spin-projected (AP) methods in modeling O_3 chemistry.

S1.2 The effect of regularizer strength in κ-OOMP2

The strength of the regularizer (κ) has been shown to strongly influence the performance of the κ-OOMP2 method, and different values of κ are appropriate for different applications.[5,9,10] In particular, like OOMP2 itself ($\kappa \to \infty$), overly weak regularizers (κ too large) have been shown to result in artificial symmetry restoration in strongly correlated systems. Stronger regularizers (lower κ values) are necessary to recover essential spin polarization that

Table S2: Effect of regularizer strength (κ) on performance of κ-OOMP2 for benchmark ozonation reactions. Empty columns for approximate projection (AP) results indicate an absence of spin polarization for all relevant species and the given method.

	Error in ΔE (kcal mol^{-1}) def2-TZVPP						ΔE_{ref} CBS CCSDT(Q)
κ	0.80		1.10		1.45		
Substrate	SC	AP	SC	AP	SC	AP	
	Ozone Addition/Insertion Barrier Heights						
C_2H_4	8.32	18.31	0.96	–	-0.52	–	3.01
C_2H_2	8.81	18.79	1.40	–	-0.10	–	7.65
HCN	10.51	20.50	2.59	–	0.58	–	17.92
HCl	13.54	23.52	6.11	–	4.16	–	20.23
NH_3	11.43	21.42	5.52	–	4.60	–	23.13
$N(CH_3)_3$	6.78	16.76	1.57	–	1.17	–	10.33
Br^-	7.19	17.17	2.48	–	2.63	–	9.73
RMSD				–		–	–
	Ozone Addition/Insertion Reaction Energies						
C_2H_4	-17.31	-7.33	-13.98	–	-5.40	–	-56.19
C_2H_2	-14.46	-4.47	-10.98	–	-2.36	–	-63.01
HCN	-9.85	0.14	-7.69	–	-0.31	–	-19.39
HCl	-3.06	6.93	-1.92	–	4.12	–	-4.99
NH_3	-7.70	2.29	-5.13	–	2.34	–	-12.13
$N(CH_3)_3$	0.10	10.08	1.56	–	7.95	–	-12.09
Br^-	7.33	17.31	3.28	–	4.39	–	5.15
RMSD				–		–	–
	$^1O_2$ Scission Reaction Energies						
O_3	-31.87	-7.62	-22.45	-8.99	-11.13	1.69	49.83
$N(CH_3)_3O_3$	-25.21	-0.96	-21.39	-7.93	-13.26	-0.45	-9.20
BrO_3^-	-17.76	6.49	-15.30	-1.84	-8.57	4.25	15.72

is associated with systems exhibiting strong correlation. At the other extreme, excessively strong regularization leads to artificial symmetry-breaking, as is well-known for mean-field Hartree-Fock (i.e. $\kappa = 0$).[10]

Our observations for O_3 and other similar molecules are that the generally recommended[5] value of $\kappa = 1.45$ results in loss of spin-polarization in the reference determinant for O_3 and related species. This behavior persists with $\kappa = 1.10$ (which has been recommended to preserve essential symmetry breaking in transition metal systems.[10]), and spin-polarization is finally obtained with $\kappa = 0.8$ (i.e. very strong regularization). Interestingly this persistence of symmetry restoration indicates that electron correlation effects in ozone (specifically at its most stable geometry) are not as strong as in strongly correlation systems such as C_{36}

where spin-polarization is recovered in κ-OOMP2 with far weaker regularization,[11] or in transition-metal containing systems.[10] In other words, the k-OOMP2 results suggest that O_3 at its equilibrium geometry is not strongly correlated because it does not exhibit *essential symmetry breaking* for κ values in the recommended range.[10]

As a corollary, the use of too-strong regularizers overly dampens the effects of dynamic correlation, and this effect is apparently significant for the O_3 systems here, such that $\kappa = 0.8$ results in poor agreement with benchmark energies (Table S2). Instead, the best agreement with CCSDT(Q) benchmarks[1] is achieved with $\kappa = 1.45$. Even still, the ωB97X-V and ωB97M-V DFAs outperform κ-OOMP2. In particular, none of the tested parameterizations of κ-OOMP2 achieve chemical accuracy for the thermodynamics of O_3 splitting ($O_3 \rightarrow {}^1O_2 + O(3\,p)$), which has been put forth as a test system for ozone modeling.[1] As a result, we do not use κ-OOMP2 for any of the main results of this paper, despite its success for other strongly correlated systems.

S1.3 Understanding spin contamination in DFA energies

The disparity in the accuracy of predictions for barrier heights and reaction energies, as well as the poor performance of κ-OOMP2, stems from changes in the extent of multireference character of various species across the ozonation PES. Stationary points corresponding to reactant states exhibit strong correlation and spin symmetry breaking due to the biradicaloid nature of O_3. In formation of a vdW complex, the electronic structure of O_3 does not change drastically, and both SC and AP schemes treat the complexation energy in a balanced way. As a result, all methods achieve chemical accuracy, *i.e.* errors less than $1\,\mathrm{kcal\,mol^{-1}}$, for predictions of ΔE_{vdW} (Table S1). The situation is materially different for computations of ΔE_{rxn}, where the reaction products do not exhibit the same multireference character as the reactants, evidenced by a lack of spin symmetry breaking on the unrestricted singlet surface across methods. This imbalance results in SC predictions of ΔE_{rxn} that are generally 7–9 $\mathrm{kcal\,mol^{-1}}$ too exothermic. The magnitude of these errors mirrors the size of AP correc-

tions for O_3, which are -7.7 and $-6.5\,\mathrm{kcal\,mol^{-1}}$ for ωB97X-V and ωB97M-V, respectively. Hence, the use of AP corrections brings results for these DFAs in closer agreement with the CCSDT(Q) results and explains why SC methods that are effective at predicting ΔE_{vdW} fail for ΔE_{rxn}.

By contrast, the AP scheme overestimates the barrier heights of ozonation reactions by a similar magnitude in the majority of cases, while the SC values generally hover around $1\,\mathrm{kcal\,mol^{-1}}$. Indeed, even sub-chemical accuracy is achieved with SC barrier heights in about half of the included systems, suggesting a balance in the degree of correlation errors in reactants and transition states is present on the SC but not the AP surfaces. It is difficult to explain this solely on the basis of spin contamination, as the transition structures for cycloaddition and linear addition are spin pure, and therefore unaffected by a spin correction. Nevertheless, SC predictions of ΔE_{TS} exhibit a similarly high degree of accuracy as they do for the insertion reactions of HCl and NH_3, where the transition structures are spin-polarized. Indeed, AP corrections uniformly reduce the accuracy of computed ΔE_{TS}, regardless of the spin-polarization of a given transition structure. These results would be unintuitive if one expected the extent of spin-polarization in DFA calculations to match those of exact wavefunction theory. However, the exact Kohn-Sham DFT orbitals are expected to be unrestricted in general,[12] and we remind the reader that the extent of spin-contamination in DFT is measured for the fictitious reference system of non-interacting electrons, rather than the physical system of interacting electrons.

S2 Conformer specifications

Our computational work utilized a number of distinct isomers and conformers for all derivatives of 4-aminodiphenylamine (4ADPA, **2**) and *N*-methyl-*N′*-phenyl-*p*-phenylenediamine (MePPD, **3**), and only results corresponding to the minimum-energy conformers are reported in the main text. We include structural details for each conformer in the molecular

coordinate (.xyz) files accompanying the SI, and energies for these in the corresponding spreadsheet. The labeling scheme for these conformers is defined in Figures S1-S4. Conformers for quinone diimine (QDI) structures follow the same ordering, though they are not explicitly included in the figures below.

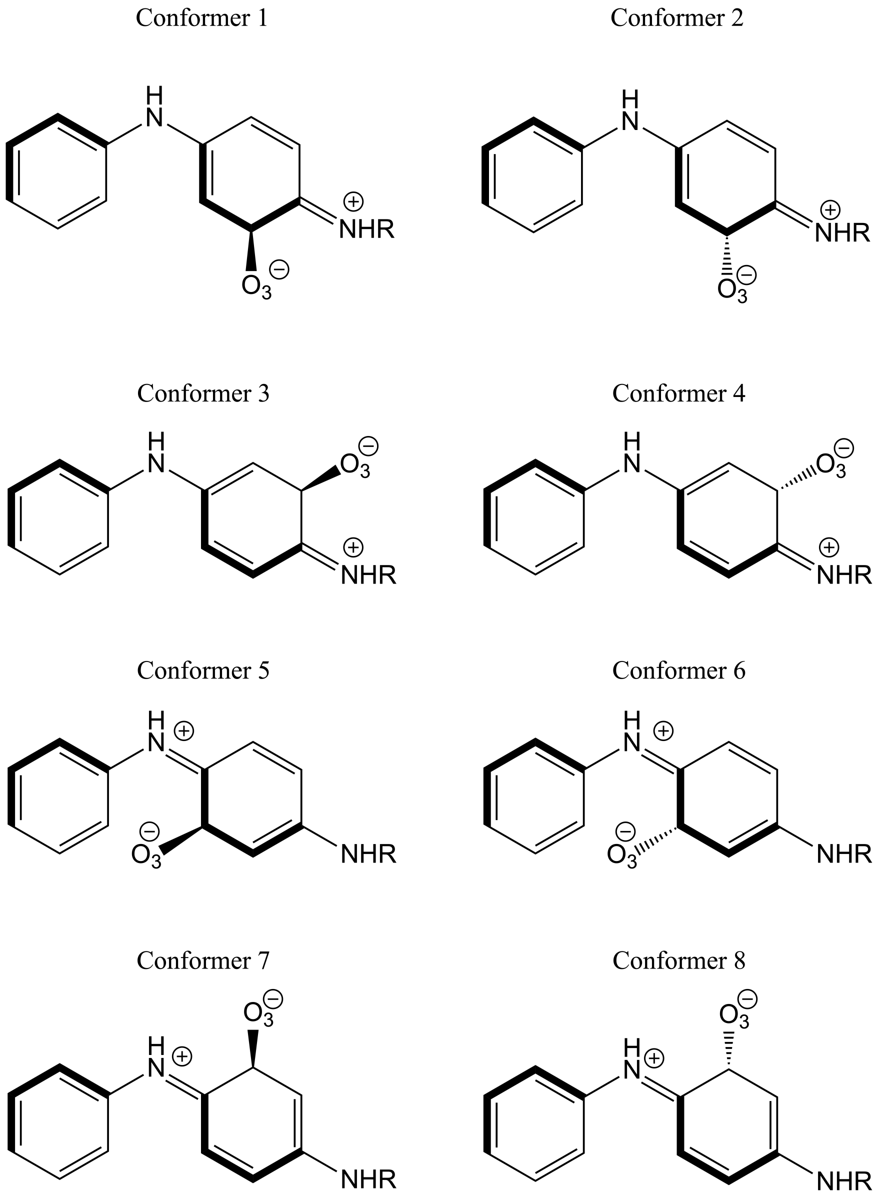

Figure S1: Definition of conformer labeling for PPD DeMore adducts. R = H (4ADPA), Me (MePPD).

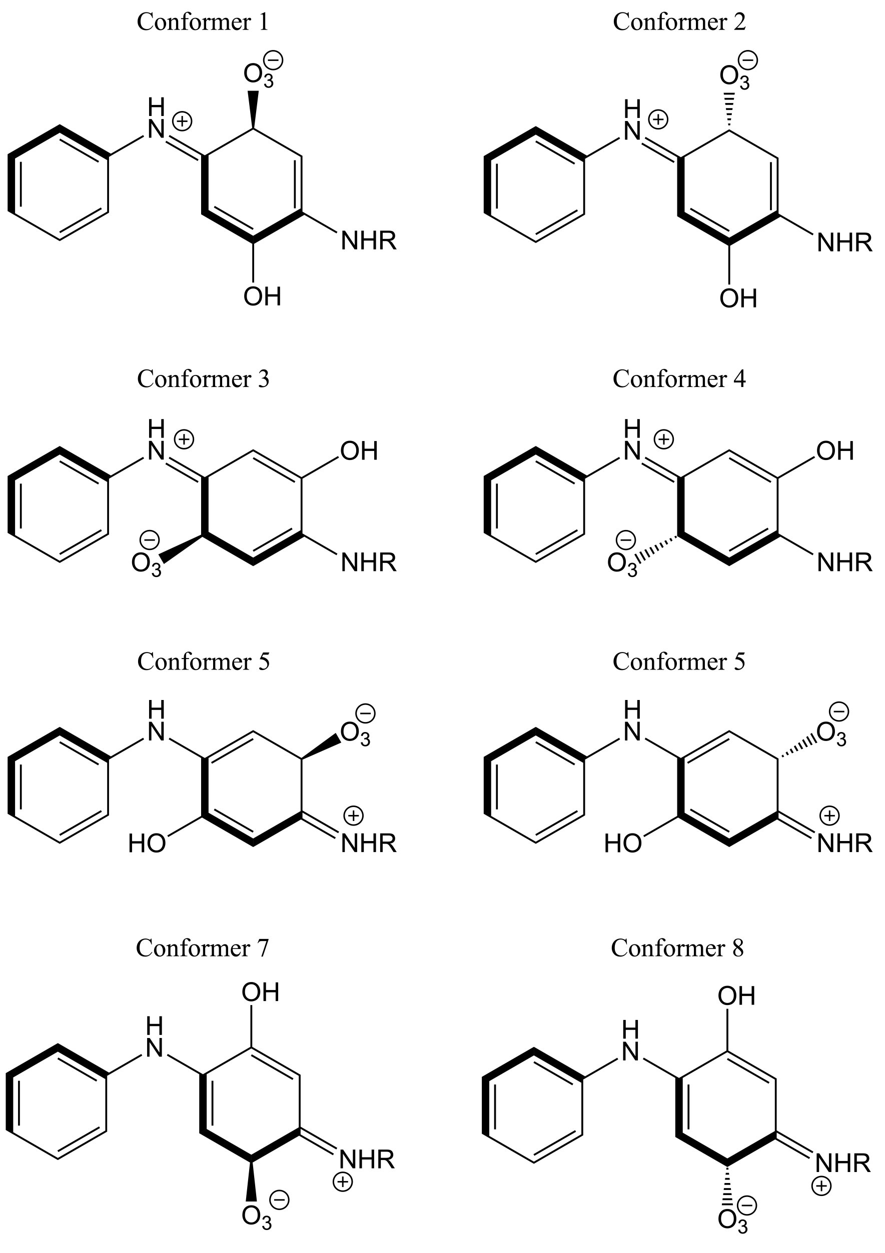

Figure S2: Definition of conformer labeling for PPD-OH DeMore adducts. R = H (4ADPA), Me (MePPD).

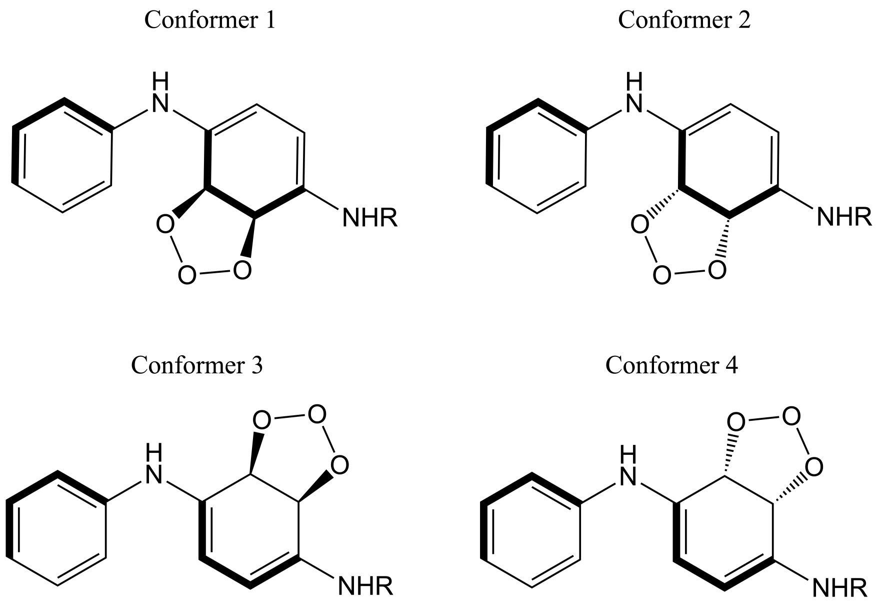

Figure S3: Definition of conformer labeling for PPD primary ozonides. R = H (4ADPA), Me (MePPD).

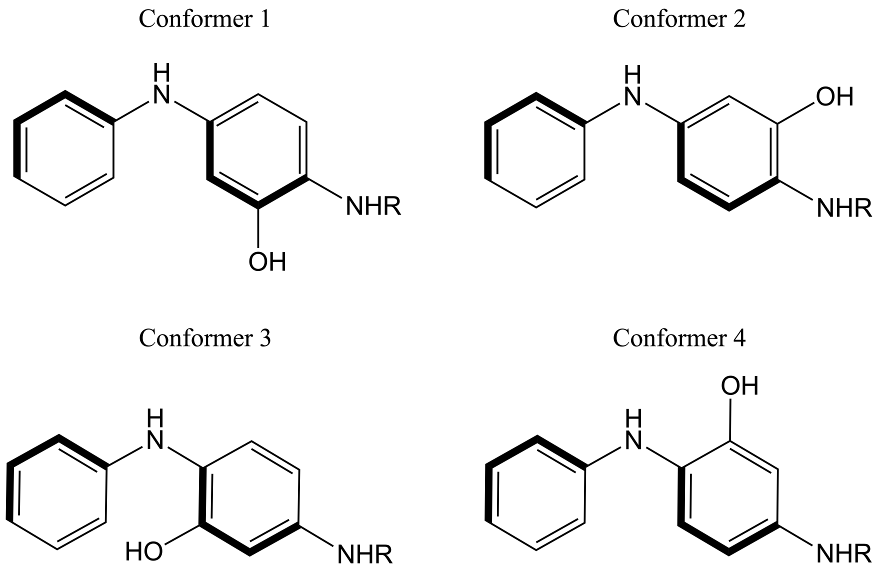

Figure S4: Definition of conformer labeling for hydroxylated PPDs. R = H (4ADPA), Me (MePPD).

References

(1) Trogolo, D.; Arey, J. S.; Tentscher, P. R. Gas-phase ozone reactions with a structurally diverse set of molecules: Barrier heights and reaction energies evaluated by coupled cluster and density functional theory calculations. *J. Phys. Chem. A* **2019**, *123*, 517–536.

(2) K.Yamaguchi,; F.Jensen,; A.Dorigo,; K.N.Houk, A spin correction procedure for unrestricted Hartree-Fock and Møller-Plesset wavefunctions for singlet diradicals and polyradicals. *Chem. Phys. Lett.* **1988**, *149*, 537–542.

(3) Mardirossian, N.; Head-Gordon, M. ωB97X-V: A 10-parameter, range-separated hybrid, generalized gradient approximation density functional with nonlocal correlation, designed by a survival-of-the-fittest strategy. *Phys. Chem. Chem. Phys.* **2014**, *16*, 9904–9924.

(4) Mardirossian, N.; Head-Gordon, M. ωB97M-V: A combinatorially optimized, range-separated hybrid, meta-GGA density functional with VV10 nonlocal correlation. *J. Chem. Phys.* **2016**, *144*, 214110.

(5) Lee, J.; Head-Gordon, M. Regularized orbital-optimized second-order Møller–Plesset perturbation theory: A reliable fifth-order-scaling electron correlation model with orbital energy dependent regularizers. *J. Chem. Theory Comput.* **2018**, *14*, 5203–5219.

(6) Boys, S.; Bernardi, F. The calculation of small molecular interactions by the differences of separate total energies. Some procedures with reduced errors. *Mol. Phys.* **1970**, *19*, 553–66.

(7) Weigend, F.; Ahlrichs, R. Balanced basis sets of split valence, triple zeta valence, and quadruple valence quality for H to Rn: Design and assessment of accuracy. *Phys. Chem. Chem. Phys.* **2005**, *7*, 3297–3305.

(8) Weigend, F.; Furche, F.; Ahlrichs, R. Gaussian basis sets of quadruple zeta valence quality for atoms H-Kr. *J. Chem. Phys.* **2003**, *119*, 12753–12762.

(9) Shee, J.; Loipersberger, M.; Hait, D.; Lee, J.; Head-Gordon, M. Revealing the nature of electron correlation in transition metal complexes with symmetry breaking and chemical intuition. *J. Chem. Phys.* **2021**, *154*, 194109.

(10) Rettig, A.; Shee, J.; Lee, J.; Head-Gordon, M. Revisiting the orbital energy-dependent regularization of the orbital-optimized second-order Møller–Plesset theory. *J. Chem. Theory Comput.* **2022**, *18*, 5382–5392.

(11) Lee, J.; Head-Gordon, M. Distinguishing artificial and essential symmetry breaking in a single determinant: Approach and application to the C 60, C 36, and C 20 fullerenes. *Phys. Chem. Chem. Phys.* **2019**, *21*, 4763–4778.

(12) Pople, J. A.; Gill, P. M.; Handy, N. C. Spin-unrestricted character of Kohn-Sham orbitals for open-shell systems. *Int. J. Quantum Chem.* **1995**, *56*, 303–305.
